# Intestinal intermediate filament polypeptides in *C. elegans*: Common and isotype-specific contributions to intestinal ultrastructure and function

**DOI:** 10.1038/s41598-020-59791-w

**Published:** 2020-02-21

**Authors:** Florian Geisler, Richard A. Coch, Christine Richardson, Martin Goldberg, Carlo Bevilacqua, Robert Prevedel, Rudolf E. Leube

**Affiliations:** 1 Institute of Molecular and Cellular Anatomy, RWTH Aachen University, Aachen, Germany; 2 School of Biological and Biomedical Sciences, Durham University, Durham, United Kingdom; 3 Cell Biology and Biophysics Unit, European Molecular Biology Laboratory, Collaboration for joint PhD degree between EMBL and Heidelberg University, Faculty of Biosciences, Heidelberg, Germany; 4 Cell Biology and Biophysics Unit, European Molecular Biology Laboratory, Heidelberg, Germany

**Keywords:** Caenorhabditis elegans, Apicobasal polarity, Intermediate filaments

## Abstract

The abundance and diversity of intermediate filaments (IFs) in the *C. elegans* intestine indicate important contributions to intestinal function and organismal wellbeing. Fluorescent IF reporters localize below the actin-rich brush border and are highly enriched in the lumen-enveloping endotube, which is attached to the *C. elegans* apical junction. Mapping intestinal viscoelasticity by contact-free Brillouin microscopy reveals that the IF-rich endotube is positioned at the interface between the stiff brush border and soft cytoplasm suggesting a mechanical buffering function to deal with the frequent luminal distortions occurring during food intake and movement. In accordance, depletion of IFB-2, IFC-2 and IFD-2 leads to intestinal lumen dilation although depletion of IFC-1, IFD-1 and IFP-1 do not. Ultrastructural analyses of loss of function mutants further show that IFC-2 mutants have a rarefied endotube and IFB-2 mutants lack an endotube altogether. Remarkably, almost all IFB-2- and IFC-2-deficient animals develop to fertile adults. But developmental retardation, reduced brood size, altered survival and increased sensitivity to microbial toxin, osmotic and oxidative stress are seen in both mutants albeit to different degrees. Taken together, we propose that individual intestinal IF polypeptides contribute in different ways to endotube morphogenesis and cooperate to cope with changing environments.

## Introduction

Surface-covering epithelia separate the hostile exterior from the body interior while facilitating regulated exchange between both compartments^1–4^. The functional dichotomy becomes particularly relevant in the intestine. While resorption and secretion have been examined in detail, much less is known about the barrier function of the intestine. Besides the tripartite junctional complex, which seals the plasma membranes of adjacent cells, and surface-covering mucus, the highly specific and polarized organization of the apical cytoplasm has caught the attention in this context. It consists of the actin-rich terminal web, which anchors and crosslinks parallel actin filaments emanating from the microvillar brush border^5,6^. The terminal web is lined below by an intermediate filament (IF)-rich layer^6^. This layer has been referred to as the “desmosomal web” in vertebrates given its dense network organization and attachment to desmosomes^7^. This arrangement is conserved through evolution with the IF-rich layer being particularly prominent in the nematode *C. elegans*^8^. There, it is referred to as the endotube, which forms a contiguous periluminal sheeth and is anchored to the composite *C. elegans apical junction* (CeAJ). Based on electron microscopic immunolocalization experiments of the IF polypeptide IFB-2^9^ it was predicted that most, if not all intestinal IF polypeptides, i.e. also IFC-1, IFC-2, IFD-1, IFD-2 and IFP-2, localize to the endotube (cf.^10^). The diversity and abundance of intestinal IF polypeptides strongly suggest an important function in intestinal physiology.

Foremost, mechanical functions have been proposed^11–13^. IFs are highly flexible and extensible but stiffen at increased strain (review in^14^). Thus, they could accommodate the strain and stresses imposed on intestinal cells by the passage of food or by muscle contraction because of movement and defecation. An indication of decreasing intestinal resilience occurring during aging may be the enlarged lumen observed in older animals. Interestingly, luminal widening is also detected in mutants with perturbed endotube organization leading to prominent cytoplasmic invaginations in some instances^12,15^.

An overall protective function of the endotube was further suggested by analyses of *sma-5* and *ifo-1* mutants, both of which had been identified in genetic screens as upstream regulators of IFB-2^12,15,16^. Inactivation of *sma-5* induces local gaps within the endotube and endotube thickening, whereas *ifo-1* inactivation leads to a collapse of the entire endotube onto the CeAJ. The dysfunctional endotube, in turn, is coupled to increased susceptibility to microbial, oxidative and osmotic stress^16^.

The goal of the current study was to investigate whether and how different IF polypeptides contribute to endotube morphogenesis and function and hereby serve as polymodal and tunable stress protectors.

## Results

### Intestinal intermediate filaments are restricted to the *C. elegans* apical junction-anchored endotube

**Figure 1:**
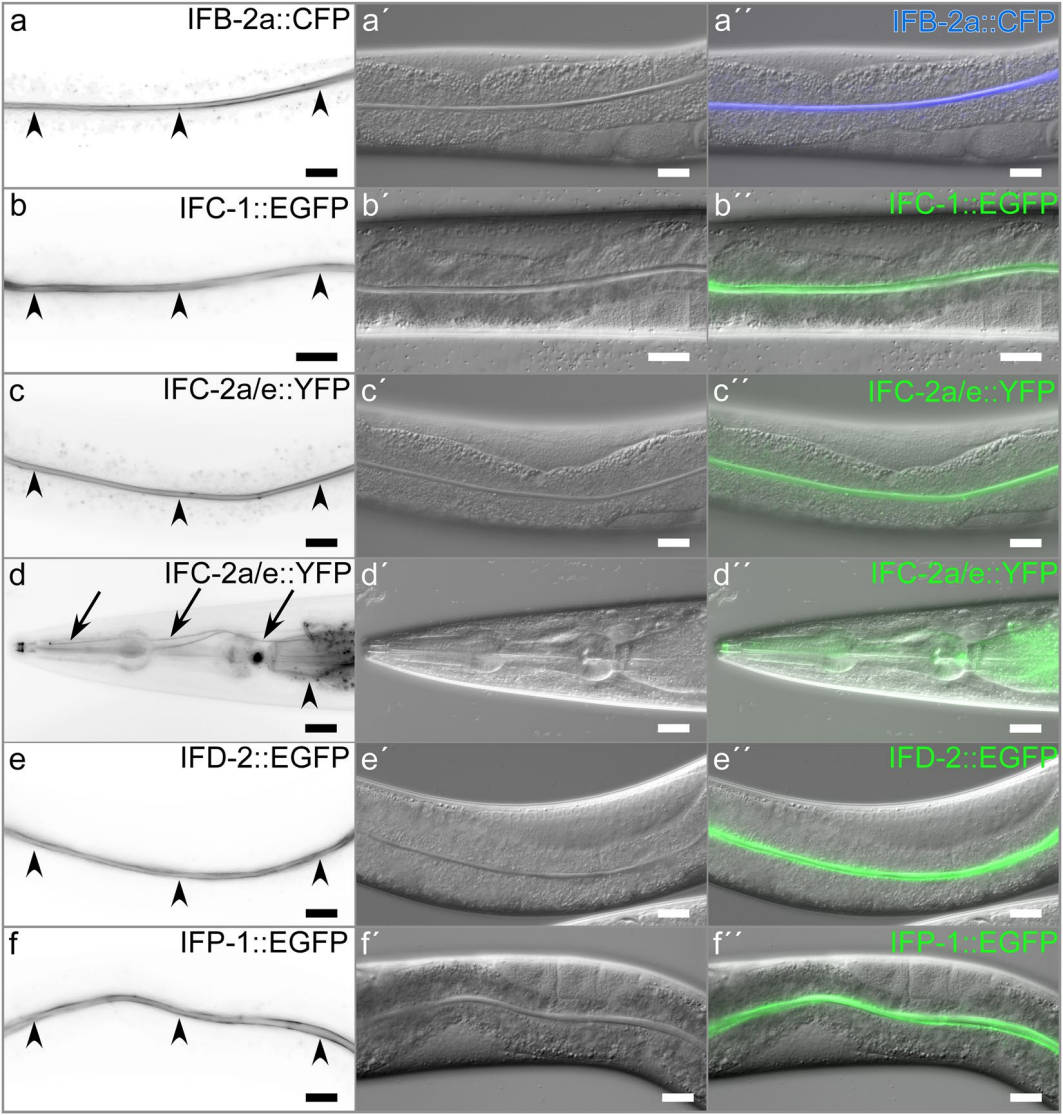
Intermediate filaments localize to the apical domain of the *C. elegans* intestine. (**a–f′′**) The microscopic images depict the distribution of fluorescent IF reporters in vital worms (fluorescence at left (inverse presentation), corresponding interference contrast in the middle, merged images at right). Note that all fluorescent reporters localize almost exclusively to the apical domain of the intestine (arrowheads). (**d–d′′**) illustrates additional non-intestinal expression of IFC-2a/e::YFP in the excretory canal (arrows in d; further detailed analyses in Supplementary Fig. S3). Scale bars: 20 µm.

**Figure 2:**
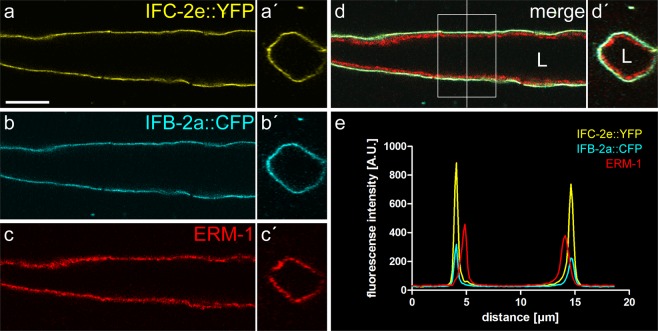
IFC-2e::YFP co-localizes with IFB-2a::CFP and both localize below ERM-1 at the apical domain of the *C. elegans* intestine. (**a–d**) Superresolution images obtained with the help of Airyscan confocal laser scanning microscopy depict the fluorescence signal of IFC-2e::YFP (**a,a′**) and IFB-2a::CFP (**b,b′**) in the methanol/acetone-fixed intestine of reporter strain BJ145 together with anti-ERM-1 immunofluorescence (**c,c′**; merged images in **d,d′**). Longitudinal single focal planes are shown in **a**–**d**, corresponding cross sections obtained along the vertical line in **d** are shown as z-reconstructions of the entire stack of 87 focal plane recordings at this position. L, lumen. Scale bar: 10 µm. (**e**) The diagram depicts the mean fluorescence profile taken across the intestinal lumen covering the boxed area in d from top to bottom (distance in µm, fluorescence intensity in arbitrary units (A.U.)).

**Figure 3:**
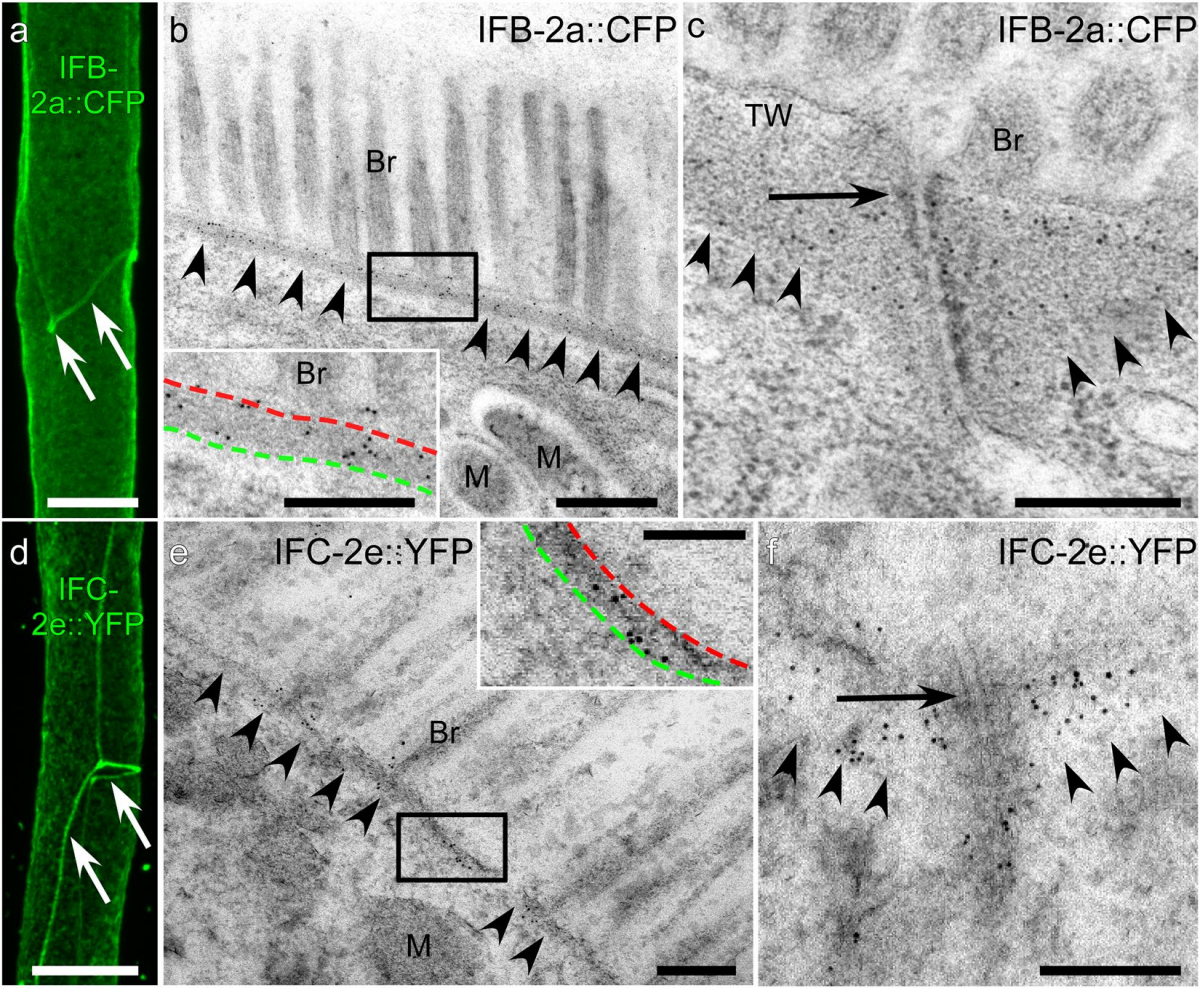
Intermediate filaments localize to the endotube and *C. elegans* apical junction in the intestine. The high resolution fluorescence confocal light microscopy images (**a,d**) and immunoelectron microscopy recordings (**b,c,e,f**) show the distribution of IFB-2a::CFP (**a–c**) and IFC-2e::YFP (**d–f**) in reporter strains BJ49 and BJ145, respectively. Note that the fluorescent polypeptides both localize to a dense periluminal network and are enriched at cell-cell junctions (arrows in **a,d**). Immunoelectron microscopy using antibodies directed against the fluorescent protein moieties (anti-GFP antibodies) in high pressure frozen specimen furthermore reveal that both intermediate filament polypeptides are mostly localized to the endotube in the subapical cytoplasm (arrowheads; framed with green and red dotted lines in insets of **b,e**) and its anchorage at the *C. elegans* apical junction (arrows in **c,f**) with some minor signal in the electron lucent terminal web (TW). Br, brush border; M, mitochondrion. Scale bars: 10 µm in **a,d**, 500 nm in (**b)**, 250 nm in inset of **b**, 250 nm in **c**, 200 nm in **e,f**, 100 nm in inset of **e**.

To examine the localization of intestinal IFs in vital worms we expanded previous reports, which employed antibodies and cDNA reporters^9,11,17–20^, by also using large fosmid constructs with *egfp*-tagged IF genes and CRISPR technology to fuse fluorescent tags onto the endogenous gene products. In each instance, almost all fluorescence localized to the apical domain of the entire intestine (Fig. 1a–c′′,e–f′′). The fluorescence patterns were virtually indistinguishable independent of whether they contained an integrated transgene coding for CFP-tagged IFB-2, an extrachromosomal fosmid containing *egfp*-tagged *ifc-1 or ifd-2*, an integrated fosmid containing an *egfp*-tagged *ifp-1*, or an engineered endogenous gene coding for YFP-tagged IFC-2. To show that the different IF polypeptides co-localize, double fluorescent reporter strain BJ145 was prepared producing carboxyterminally-labelled IFC-2 and IFB-2. Superresolution Airyscan confocal laser scanning microscopy revealed that both co-localize precisely and exclusively at the apical domain of the intestine (Fig. 2). Comparing their localization with that of the actin-binding protein ERM-1 furthermore demonstrated that the IFs are positioned slightly below ERM-1 (Fig. 2). *En face* views further revealed that the fluorescent IFB-2 and IFC-2 reporters are part of dense filamentous networks, which abut at adjacent cell borders (Fig. 3a,d). Immunoelectron microscopy was performed to further specify the localization of the fluorescent intestinal IF polypeptides. Figure 3b,e show that the IFB-2 and IFC-2 immunosignals were detectable throughout the electron dense endotube. A few gold particles were also present in the electronlucent terminal web region above and in proximity to the microvillar actin rootlets. The cytoplasm was virtually devoid of immunosignal. In accordance with the light microscopic observations, IFB-2 and IFC-2 were also detectable along the entire CeAJ (Fig. 3c,f). These data are in good agreement with earlier immunoelectron microscopic data on IFB-2 distribution^9^.

While the expression of IFB-2, IFC-1, IFD-1, IFD-2 and IFP-1 is mostly, if not completely restricted to the intestine (this study and^18^), the expression of IFC-2 is more complex. Previous reports stressed the strong intestinal expression as detected by antibody staining and extrachromosomal reporter constructs^11,17^. Yet, the data in Fig. 1d together with other recent analyses provide conclusive evidence for expression at other sites^20–22^. The situation is further complicated by alternative transcriptional units at the *ifc-2* gene locus (Supplementary Fig. S1). The WormBase release WS272 lists four gene models. They encode 1128 amino acid-long IFC-2a and 1248 amino acid-long IFC-2b differing only at their carboxyterminal 15 and 135 amino acids, respectively. Both isoforms, which have also been referred to as EXC-2a and EXC-2b^21^, consist of an aminoterminal half, that is not related to IFs and a typical IF-moiety at their carboxyterminal half. Isoform IFC-2c (685 amino acids), which has been named ECP-1 by others^20^, corresponds to the aminoterminal half of IFC-2a/b. It is encoded by exons 1–10 and contains a unique 45 amino acid-long carboxyterminus. The 622 amino acid-long protein IFC-2d is generated from a transcript that is produced from an alternative promoter located in intron 10 of the *ifc-2a*/*ifc-2b* gene^20^. It encodes the IF-moiety of IFC-2b and contains a short 14 amino acid-long aminoterminal extension. It was previously termed c2H^23^. Work by Dodemont *et al*.^23^ further suggested that another IF variant (referred to as c2L) exists which is not listed in WormBase. It is 502 aminoacids long, corresponds to the IF moiety of IFC-2a and shares the 14 aminoterminal amino acids with IFC-2d. The existence of the encoded polypeptide is supported by the observations in Fig. 1c–d′′ (further discussion below). We propose to refer to it as IFC-2e.

Taken together, we conclude that the *ifc-2* gene locus contains two separate transcriptional units with promoter 1 driving the expression of *ifc-2a/b/c* and promoter 2 driving the expression of *ifc-2d/e*. Both promoters have been shown to induce GFP-reporter expression^20^. Furthermore, Northern blot experiments using a probe specific for the IF-encoding moiety detected two minor large transcripts and at least one major small transcript, which would account for the alternative transcripts *ifc-2a/b* and *ifc-2d/e* from the two different promoters. In accordance, Al-Hashimi *et al*.^21^ identified polypeptides corresponding in size to IFC-2a and IFC-2b but not to IFC-2c in anti-GFP immunoblots of knock-in strain BK531 producing an aminoterminally 3xFLAG- and GFP-tagged IFC-2a/b/c reporter. Anti-GFP immunoblotting of our knock-in strain BJ316 encoding carboxyterminally YFP-tagged IFC-2a/e, on the other hand, showed a weak band expected for the IFC-2a::YFP fusion protein and a much stronger band for the expected IFC-2e::YFP fusion protein (Supplementary Fig. S2). Of note, the IFB-2 reporter, which is expressed at a comparable level to the endogenous gene (not shown), is much more abundant than IFC-2e.

The two *ifc-2* promoters also confer differential cell-type specific expression. While promoter 1 is inactive in the intestine, promoter 2 is highly active^20^. This is in agreement with the observed lack of aminoterminally tagged endogenous IFC-2a/b/c and strong expression of carboxyterminally tagged endogenous IFC-2a/e in the intestine (^21^ and Supplementary Fig. S3). Antibody staining further corroborated this conclusion^20^. The relative levels of IFC-2d vs. IFC-2e are currently unknown but RNAseq data in WormBase suggest high abundance of *ifc-2e* vs. low levels of *ifc-2d* mRNA. Since promoter 2 is exclusively active in the intestine^20^, promoter 1 must be the sole source for *ifc-2* gene expression in non-intestinal cells. To deduce information on isotype expression in these cells, we side-by-side compared the fluorescence in knock-in strain BJ316 producing carboxyterminally-tagged IFC-2a/e with that in knock-in strain BK531 producing aminoterminally-tagged IFC-2a/b/c (Supplementary Fig. S3; see also^21^). As expected, the intestine was fluorescent in BJ316 but not in BK531. Comparable fluorescence signals were observed in the excretory canal, pharyngeal posterior bulb, pharyngeal-intestinal valve and interfacial uterine cells in both strains indicating expression of IFC-2a with little or no IFC-2b/c contribution. On the other hand, the stronger fluorescence signal in BK531 in the pharyngeal corpus suggests substantial expression of IFC-2b/c (which are not detected in BJ316). Since the intestinal-rectal valve was only positive in BK531 but not in BJ316, we concluded that IFC-2b/c are the major if not sole isoforms in this cell. These conclusions are, except for the interfacial uterine cells, supported by immunostaining with antibodies directed against the aminoterminal IFC-2c moiety^20^.

Taken together, we conclude that IFC-2a and IFC-2b are the primary if not sole isoforms in the excretory canal, pharyngeal posterior bulb, pharyngeal intestinal valve and interfacial uterine cells. IFC-2b/c are the predominant isoforms in the pharyngeal corpus with little IFC-2a production and are most likely the sole isoforms in the intestinal rectal valve. Note, that there is no conclusive evidence for the existence of isoform IFC-2c since Northern blots and immunoblots have failed to detect this isoform so far. Antibodies directed against the predicted unique IFC-2c carboxyterminus may help to clarify this issue. Finally, we can state that IFC-2d and IFC-2e are synthesized in the intestine at low and high levels, respectively.

### Intestinal intermediate filaments are positioned at the interface between the stiff microvillar brush border and the comparatively soft cytoplasm

**Figure 4:**
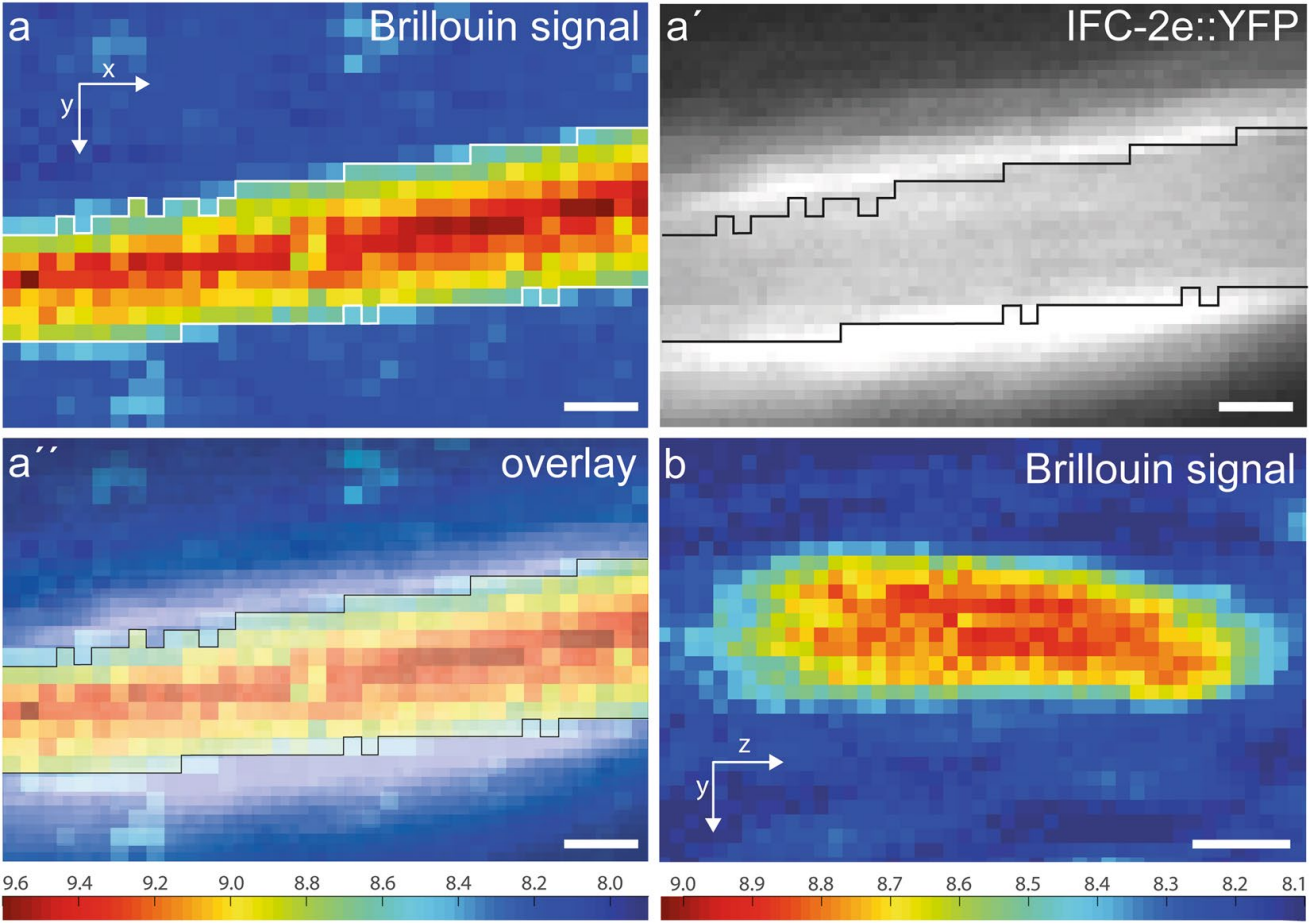
Intermediate filaments localize to the transition zone between regions with different viscoelastic properties. Brillouin microscopy was performed to examine viscoelastic properties of the apical domain of intestinal cells. (**a,b**) show projection views of the Brillouin frequency shift recorded in a longitudinal (**a**) and transverse (**b**) plane of the intestinal lumen and the surrounding cytoplasm of two living worms. Comparison with the IFC-2e::YFP fluorescence (**a′**) and superposition of both signals (**a′′**) shows that the intermediate filament-rich endotube separates the apical compartment with high stiffness from the softer cytoplasmic compartment below. Scale bars: 2 µm. Calibration bars (units in GHz) at bottom left refers to a, at bottom right to b.

**Figure 5:**
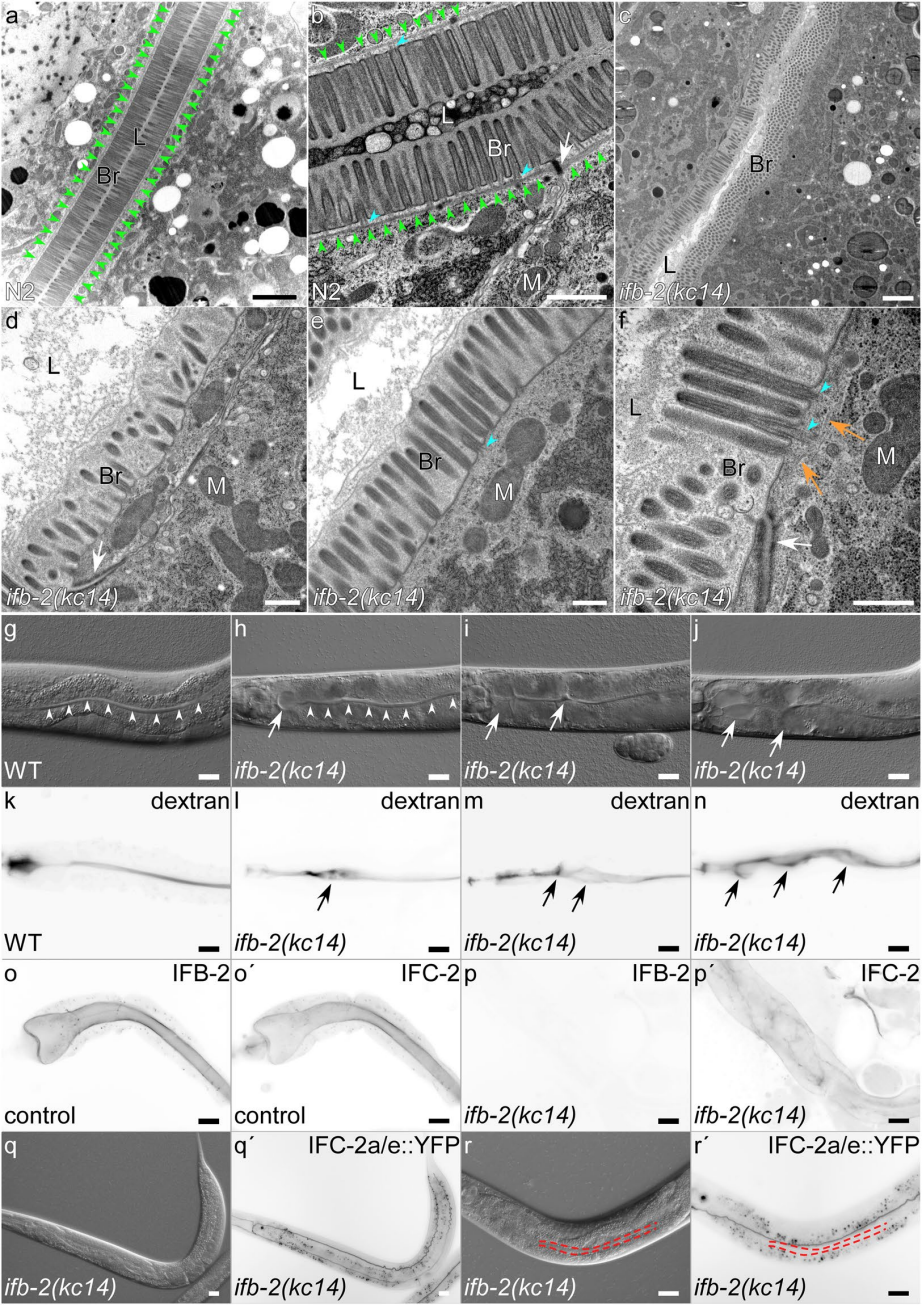
Knockout of IFB-2 prevents intestinal endotube formation and leads to cytoplasmic mislocalization of IFC-2a/e in the intestine but not in the excretory canal. (**a–f**) Electron micrographs of wild-type (**a,b**) and *ifb-2(kc14)* knockout intestines (**c–f**). The electron-dense periluminal endotube (green arrowheads in **a,b**) and its association with the rootlets of the microvillar actin filament bundles (blue arrowheads in **b**) is readily seen in the wild-type intestine but is not detectable in the mutant intestine. Endotube loss is associated with mild and locally variable defects in microvillar orientation (**d–f**) with actin filament bundles still protruding into the apical cytoplasm (blue arrowheads in **e,f**). L, lumen; Br, brush border; M, mitochondrion. (**g–n**) The interference contrast images and fluorescence images of ingested dextran show luminal alterations in *ifb-2(kc14)* adults (**h–j,l–n**) in comparison to the wild type (**g,k**). Note the presence of extensive luminal widenings (arrows) in the anterior intestine that seem to spread toward the posterior. (**o–p′**) The immunofluorescence images show the distribution of IFB-2 as detected by anti-IFB-2 antibodies (**o,p**) and of IFC-2 as detected by anti-IFC-2 antibodies (**o′,p′**) in isolated intestines of control (**o,o′**) and *ifb-2(kc14)* knockout animals (**p,p′**). IFB-2 and IFC-2 display a smooth periluminal staining in control intestines while no IFB-2 signal can be detected in the mutant intestine and apical IFC-2 localization is lost with weak but increased cytoplasmic staining. **(q–r′**) Microscopy of living *ifb-2(kc14)* mutants shows the distribution of the IFC-2a/e::YFP reporter encoded by the engineered *yfp*-tagged *ifc-2* allele *kc16* (fluorescence images in **q′, r′**; corresponding interference contrast images in **q,r**) further confirming loss of apical IFC-2 in the intestine (intestinal lumen marked with red dotted line in **r–r′**), while localization in the excretory canal remains unaffected. Scale bars: 2 µm in (**a**,**c)**; 500 nm in (**b,d–f)**; 20 µm in (**g-r′)**.

Hypothesizing that the precise positioning of the IF-rich endotube serves an important mechanical function, we performed Brillouin microscopy. This method allows the contact-free determination of viscoelastic properties in living cells and tissues at subcellular resolution^24–26^. To this end, IFC-2e::YFP reporter worms were immobilized by 10% levamisole prior to imaging. The Brillouin signal, i.e. the Brillouin frequency shift that is a proxy for stiffness^25,26^, revealed two regions: a central area of high stiffness that is surrounded by a region of low stiffness (yellow to red versus blue in Fig. 4a,b). Superposition of the IFC-2e::YFP fluorescence signal showed that the IF layer is located at the transition zone between the stiff and soft region (Fig. 4a′,a′′). We therefore conclude that the region of high stiffness corresponds to the apical domain, which is composed of microvilli containing bundled and membrane-bound actin filaments (for increased stiffness of F-actin bundles see, e.g.^27^). On the other hand, the region of low stiffness corresponds to the less well organized cytoplasm below. Since the region of the endotube-restricted IF polypeptides is few tens of nanometers in thickness only as measured by electron microscopy (62.29 ± 9.237 nm, n = 14; see also Fig. 5a,b), it cannot be resolved by Brillouin microscopy. We hypothesize, however, that the highly flexible and extensible IF layer acts as a shock absorber between the region of high and low stiffness.

### IFB-2 knockout prevents endotube formation and induces IFC-2 redistribution

To study the contribution of IFB-2 to IF network formation in the *C. elegans* intestine, CRISPR/cas9-mediated gene inactivation was performed. Disrupting the open reading frame of *ifb-2* in codon 14 by introduction of a thymidine leads to a frameshift with a stop codon in exon 2 at codon position 30 (Supplementary Fig. S4a). Identical mutations were induced in the endogenous gene and the integrated fluorescent reporter of strain BJ49 that was used for mutagenesis. These mutations prevented endotube formation (Figs. 5a–f, S4a; see also^16^). Despite the absence of the electron dense endotube, the morphology of the adjacent microvillar brush border appeared to be only mildly affected presenting slightly disordered microvilli. The degree of disorder, however, differed regionally (compare, e.g., Fig. 5d,e). Microvillar actin bundles looked normal extending into the apical cytoplasm. The emerging actin rootlets, however, were often in close apposition to microtubules (orange arrows in Fig. 5f). The CeAJ was still clearly detectable but contained less apposed electron dense plaque material (compare white arrows in Fig. 5b with Fig. 5d,f). The intestinal lumen was slightly enlarged and irregular as judged by electron microscopy (compare Fig. 5a with Fig. 5c) and interference contrast light microscopy (compare Fig. 5g with Fig. 5h–j). In addition, large local dilations were frequently observed that were mostly detected in the anterior intestine often extending toward the posterior (Fig. 5h–j). To assess the luminal alterations more reliably, worms were fed with fluorescent dextran (Fig. 5k–n). This showed that luminal width was not affected in L1-L3 larvae. Minor dilations were spuriously detected in L4 larvae and young adults. In 11 of 19 three days old animals luminal widening was clearly detectable increasing to 13 of 14 four days old animals.

Immunofluorescence microscopy was performed next to study the effect of IFB-2 depletion on other intestinal IFs. Figure 5o–p′ shows the complete absence of IFB-2 and the loss of apical IFC-2, which was distributed for the most part in a non-filamentous diffuse state throughout the cytoplasm. These findings were further corroborated in IFB-2 knockout worms carrying the IFC-2a/e::YFP-encoding allele *kc16*. In this instance, the intestinal IFC-2e::YFP was virtually absent while the non-intestinal IFC-2a::YFP was still prominent in the excretory canal (Fig. 5q–r′).

### IFC-2 knockout induces endotube thinning and cytoplasmic re-localization of IFB-2 with luminal widening but does not reproduce the previously reported cytoplasmic invagination phenotype

**Figure 6:**
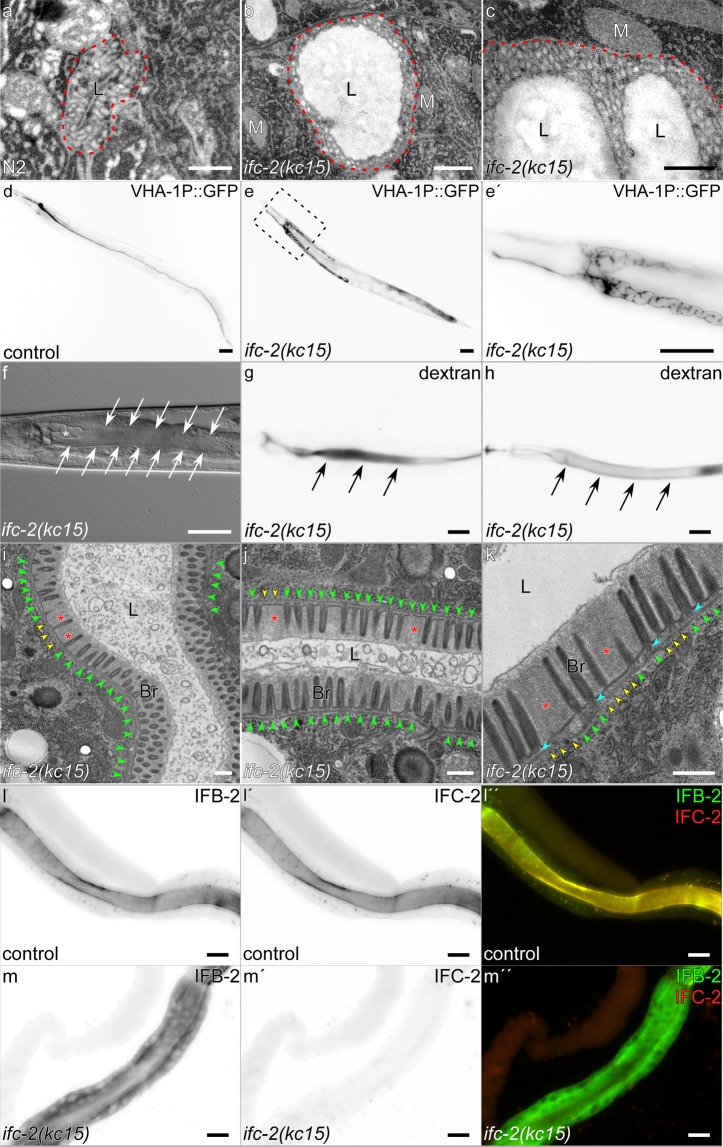
Knockout of IFC-2 leads to severe excretory canal defects and luminal widening of the intestine with cytoplasmic mislocalization of IFB-2. (**a–c**) Electron micrographs of wild-type and *ifc-2(kc15)* mutant excretory canals. Note the massively dilated lumen (L) in the mutant compared to control animals (excretory canal surrounded by canaliculi demarcated by red dotted line). M, mitochondrion. (**d–e′**) Microscopy of worms producing GFP controlled by the excretory canal-specific promotor VHA-1P either in a wild-type background (**d**) or in an *ifc-2(kc15)* mutant (**e**; higher magnification in **e′**). Note the shortened, thickened and tortuous lumen of the mutant excretory canal. (**f**) Differential interference contrast image of an *ifc-2(kc15)* mutant depicting shortened and dilated excretory canal with prominent cysts (asterisk) and the substantially dilated intestine (arrows). (**g,h**) Fluorescence microscopy of ingested fluorescent dextran in the intestine of adult *ifc-2(kc15)* mutants depicting luminal widening (arrows; control in Fig. 5k). (**i–k**) The electron micrographs depict partial thinning of the electron-dense periluminal endotube (yellow versus green arrowheads). Microvillar actin filament bundles anchoring at the endotube are still discernible (blue arrowheads in **k**) but microvillus spacing is increased often in regions with thinner endotube (red asterisks in **i–k**). L, lumen; Br, brush border. (**l–m′′**) The immunofluorescence images show the distribution of IFB-2 (**l,m**) and IFC-2 (**l′,m′**; merged images in **l′′,m′′**) in isolated intestines of either control (**l–l′′**) or *ifc-2(kc15)* mutant animals (**m-m′′**). In control intestines, IFB-2 and IFC-2 display a smooth periluminal staining, while no IFC-2 signal can be detected in *ifc-2(kc15)*. This is accompanied by substantial loss of apical IFB-2 localization. Scale bars: 500 nm in (**a-c,i–k)**; 50 µm in **d,e**; 40 µm in **f**; 20 µm in (**e′,g,h,l–m′′)**.

To examine the effect of IFC-2 on the ultrastructure of the apical membrane domain of intestinal cells, the *ifc-2* gene was inactivated. This was done by CRISPR/cas9 technology in the *kc16[ifc-2a/e*:*:yfp]* background. The resulting *ifc-2* allele *kc15* carries a 32 bp deletion in exon 11 leading to a frameshift with a stop codon after 11 amino acids (ICWSHLHNPNP; Supplementary Fig. S4b). The mutant allele encodes carboxyterminally mutated and truncated mutants IFC-2a/b (660 amino acids) and IFC-2d/e (34 amino acids) but does not affect IFC-2c production. Production of the IF-moiety should therefore be efficiently abrogated throughout the mutant worms. As to be expected from results of other laboratories using comparable gene inactivation approaches^20–22^, the lumen of the excretory canal was considerably enlarged with alterations of the apical domain in this mutant (Fig. 6a–c), which was confirmed by light microscopic analyses (Fig. 6d–f). In the intestine, however, an endotube-like structure was clearly discernible in *ifc-2(kc15)* animals (Fig. 6i–k). But the endotube was thinner than in the wild type and exhibited local rarefication (N2: 62.29 ± 9.237 nm, n = 14; *ifc-2(kc15)*: 44.10 ± 7.324 nm, n = 39; p < 0.0001). Microvilli presented an overall normal morphology. Yet, microvillar spacing was irregular with multiple gaps disrupting the typical tightly-arranged brush border. Luminal enlargement was seen frequently but was much less prominent than would have been expected from our light microscopic analyses of *ifc-2 RNAi* treated worms^11^. We could not detect the previously reported conspicuous cytoplasmic invaginations. Even using the identical reagents described in that publication^11^, we could not reproduce the phenotype. Our only explanation is that the different observations are due to the different lab environments in Mainz and Düsseldorf where the previous experiments were performed versus RWTH Aachen University where the more recent experiments were performed.

To assess luminal width alterations in *ifc-2(kc15)* animals interference contrast images were recorded and fluorescent dextran feeding experiments were done (Fig. 6g–h). The phenotype could only be detected in young adult animals onwards. Luminal widening was noted in 7 of 10 three days old and 10 of 13 four days old mutants upon dextran feeding. Staining of dissected *ifc-2(kc15)* intestines with anti-IFB-antibodies furthermore revealed a significant loss of apical IFB-2 with concurrent increased non-filamentous cytoplasmic IFB-2 (Fig. 61–m′).

### IFC-1, IFD-1 and IFP-1 are not essential for intestinal lumen maintenance whereas loss of IFD-2 induces cytoplasmic invaginations

In the next set of experiments all other intestinal IFs were downregulated. First, IFC-1 was reduced by RNAi in IFC-1::EGFP reporters. In spite of complete loss of IFC-1::EGFP fluorescence intestinal lumina were still unaffected as judged by interference contrast microscopy (Supplementary Fig. S5a–c′′) and IFB-2a::CFP distribution was not affected (Supplementary Fig. S5c–c′′). Examination of loss-of-function *ifd-1* and *ifd-2* alleles elicited two different phenotypes: *ifd-1(ok2404)* did not visibly affect the lumen and the distribution of either IFC-2 or IFB-2 whereas *ifd-2(bz187)* induced luminal widening with cytoplasmic invaginations (Supplementary Fig. S6). Of note, IFC-2 and IFB-2 were still localized to the apical domain in the latter mutant. Using CRISPR/cas9 technology the native *ifp-1* gene was mutated introducing a frame shift immediately after the start codon thereby creating a stop codon in position 6 (*ifp-1(kc18)*, Supplementary Fig. S4c). In parallel, integrated *ifp-1* reporter gene (*kcIs40[ifp-1::ifp-1::egfp*]) was mutated by introducing a frameshift causing a stop codon in position 52 (kcIs41[ifp-1p::ifp-1[16-19del]::egfp *kcIs40], Supplementary Fig. S4d). The mutations affected neither intestinal lumen width nor the distribution of IFB-2 (Supplementary Fig. S7a–d). The results were further corroborated in *ifp-1(RNAi)* experiments (Supplementary Fig. S7e–f′′). Given the phenotypic complexity, we decided to carry out all further experiments only for IFB-2 and IFC-2 mutants.

### Depletion of either IFB-2 or IFC-2 affects the actin cytoskeleton and its associated linker plastin 1 differently

**Figure 7:**
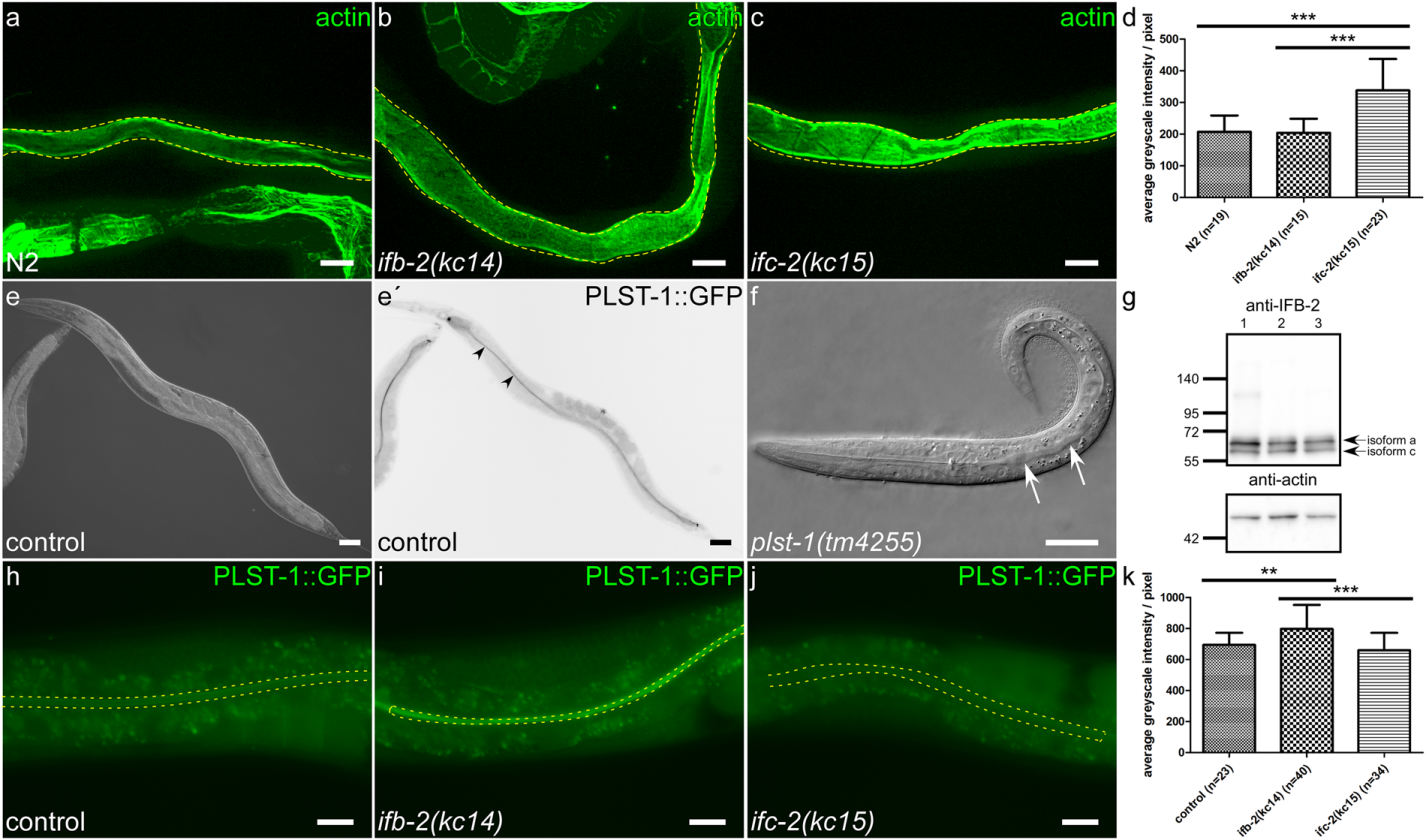
IFC-2 and IFB-2 interact with actin and the actin bundling protein PLST-1 in an isotype-specific fashion. (**a–c**) The maximum intensity fluorescence images show staining of actin filaments with Alexa Fluor 488-conjugated phalloidin (wild-type N2 in a, *ifb-2(kc14)* in **b** and *ifc-2(kc15)* in **c**). Areas demarcated by yellow dotted lines were used for quantification, which is shown in (**d**). The expression of actin is significantly increased in *ifc-2(kc15)* compared to *ifb-2(kc14)* and wild-type N2 (N2: 207 ± 51, n = 19; *ifb-2(kc14)*: 203 ± 44, n = 15; *ifc-2(kc15)*: 338 ± 99, n = 23; two independent experiments). (**e,e′**) The corresponding images show the interference contrast recording (**e**) and fluorescence micrograph detecting PLST-1::GFP produced from *plst-1* allele *msn190*. Note the periluminal PLST-1::GFP enrichment throughout the intestine (arrowheads). (**f**) shows an interference contrast image of a larva harboring the loss-of-function *plst-1* allele *tm4255*. Regions of dilated intestine are marked by arrows. (**g**) The immunoblot detects IFB-2 isoforms a and c and actin in lysates of *plst-1(tm4255)* animals either with dilated intestinal lumen (lane 1) or without (lane 2) and in lysates of wild-type N2 (lane 3). The immunosignal in the mutant animals with lumen dilation is increased coincident with a shift from IFB-2c to IFB-2a. The position and size in kDa of co-electrophoresed molecular weight markers are shown at left. (**h–k**) shows single plane *in vivo* images of PLST-1::GFP fluorescence produced from the tagged endogenous *plst-1* gene in wild-type (**h**), *ifb-2(kc14)* (**i**) and *ifc-2(kc15)* (**j**) backgrounds. The areas delineated by yellow dotted lines were used for quantification, the results of which are depicted in (**k**). Loss of IFB-2 induces higher expression of PLST-1 in the apical intestine, whereas loss of IFC-2 does not (N2: 694 ± 78, n = 23; *ifb-2(kc14)*: 796 ± 155, n = 40; *ifc-2(kc15)*: 660 ± 111; n = 34; two independent experiments). Significance: **p < 0.01, ***p < 0.001. Scale bars: 20 µm.

Based on the previous observation that the intestinal filament organizer IFO-1 affects the distribution of intestinal IFs and actin^15^, we wanted to find out whether loss of IFs also perturbs the apical enrichment of actin in intestinal cells. By quantifying phalloidin staining no differences could be detected between wild type and *ifb-2(kc14)*. On the other hand, fluorescence was significantly increased in *ifc-2(kc15)* (Fig. 7a–d).

It has been reported that the intestinal actin and IF systems are connected through the actin-bundling protein plastin 1 in mouse^28^. In agreement with previous observations^29^ we detected periluminal enrichment of PLST-1::GFP in the entire intestine of a *C. elegans* strain carrying knock-in allele *msn190[plst-1::gfp]* (Fig. 7e,e′). Remarkably, the *plst-1* loss-of-function allele *tm4255*^29^ induced luminal dilation in some but not all larvae (Fig. 7f). Assessment of IFB-2 expression by immunoblotting revealed that it was increased in *tm4255* mutant animals with dilated intestinal lumen compared to those without dilated lumen and the wild type (Fig. 7g). The overall increase in IFB-2 was accompanied by a shift from the smaller IFB-2c variant to the larger IFB-2a variant as was reported for N2 subjected to microbial toxin stress^16^. Conversely, IFB-2 depletion increased PLST-1::GFP fluorescence (Fig. 7h,i,k). On the other hand, IFC-2 depletion had no measurable effect (Fig. 7h,j,k).

### Depletion of intestinal intermediate filaments affects time of development, brood size and life span

**Figure 8:**
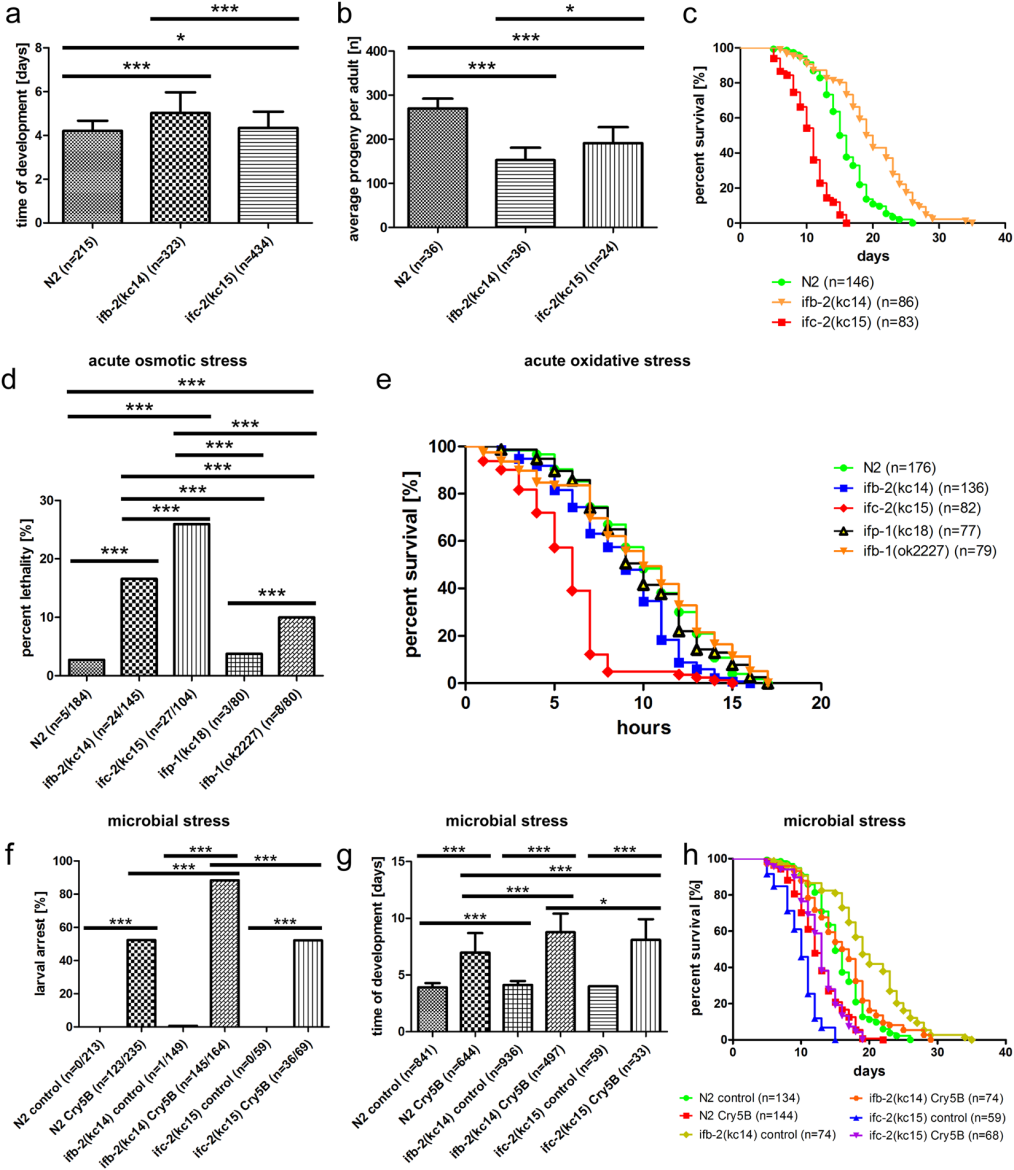
Knockout of IFB-2 and IFC-2 procrastinates development, reduces progeny, changes life span and increases stress sensitivity. (**a**) Time of development is increased in *ifb-2(kc14)* and *ifc-2(kc15)* (N2: 4.21 ± 0.46 days, *ifb-2(kc14)*: 5.03 ± 0.94 days, *ifc-2(kc15)*: 4.34 ± 0.74 days). (**b**) Adult *ifb-2(kc14)* and *ifc-2(kc15)* have reduced offspring (N2: 270 ± 22, *ifb-2(kc14)*: 153 ± 28, *ifc-2(kc15)*: 191 ± 36). (**c**) Life span analyses reveal longer median survival of *ifb-2(kc14)* and shorter median survival of *ifc-2(kc15)* compared to N2 (N2: 15.5 days, *ifb-2(kc14)*: 19.5 days, *ifc-2(kc15)*: 11 days; p < 0.0001 for N2 versus *ifb-2(kc14)* and *ifc-2(kc15)*, and for *ifb-2(kc14)* versus *ifc-2(kc15)*). (**d**) Acute osmotic stress has the strongest effect on lethality in *ifc-2(kc15)* followed by *ifb-2(kc14)* and trailed by *ifp-1(kc18)* and N2. Note that the excretory canal-specific *ifb-1(ok2227)* is intermediate between N2 and *ifc-2(kc15)* (N2: 2.72%; *ifp-1(kc18)*: 3.75%; *ifb-2(kc14)*: 16.55%; *ifc-2(kc15)*: 25.96%; *ifb-1(ok2227)*: 10.00%). (**e**) Acute oxidative stress reduces median survival in *ifb-2(kc14)* and *ifc-2(kc15)* but not in *ifb-1(ok2227)* (N2: 10 h, *ifp-1(kc18)*: 10 h, *ifb-2(kc14)*: 9 h, *ifc-2(kc15)*: 6 h, *ifb-1(ok2227)*: 10 h; p < 0.001 for N2 versus *ifb-2(kc14)* and *ifc-2(kc15)* and for *ifb-2(kc14)* versus *ifc-2(kc15)*; p > 0.05 for N2 versus *ifb-1(ok2227) and ifp-1(kc18))*. (**f**) Larval arrest is observed in 0.67% *ifb-2(kc14)* and not at all in N2 and in *ifc-2(kc15)* grown on control bacteria but in 88.41% of *ifb-2(kc14)*, 52.17% of *ifc-2(kc15)* and 52.34% of N2 grown on Cry5B-producing bacteria. (**g**) Cry5b prolongs time of development by 4.65 days in *ifb-2(kc14)* (control bacteria*:* 4.12 ± 0.35 days versus Cry5B-synthesizing bacteria: 8.77 ± 1.64 days), by 4.09 days in *ifc-2(kc15)* (control bacteria: 4.00 ± 0.10 days versus Cry5B-synthesizing bacteria: 8.09 ± 1.83 days) but only by 3.06 days in N2 (3.91 ± 0.37 days on control bacteria versus 6.97 ± 1.72 days on Cry5B-producing bacteria). (**h**) Life span analysis shows that *ifb-2(kc14)* survive longer and that *ifc-2(kc15)* die earlier than N2 in control conditions (19 days for *ifb-2(kc14)*; 10 days for *ifc-2(kc15)*; 15 days for N2; p < 0.0001). The life span of *ifb-2(kc14)* is severely reduced on Cry5B-producing bacteria (*ifb-2(kc14)*: 16.5 days, N2: 12 days; p = 0.0004), whereas that of *ifc-2(kc15)* is even slightly prolonged (13 days; p < 0.0001). *p < 0.05, ***p < 0.001.

Next, we wanted to study the consequences of IFB-2 and IFC-2 on the overall physiology of the worm. As expected, embryonic lethality was unaffected in *ifb-2(kc14)* and *ifc-2(kc15)* mutants underlining the exclusively postembryonic function of single intestinal IFs. Furthermore, larval arrest was only slightly increased in these mutants (N2: 0%, n = 0/225); *ifb-2(kc14)*: 0.62%, n = 1/161; *ifc-2(kc15)*: 2.41%, n = 2/83). Yet, time of development was significantly increased in both mutants with only a slight increase in *ifc-2(kc15)* and a substantial increase in *ifb-2(kc14)* (Fig. 8a). Similarly, brood size was more reduced in *ifb-2(kc14)* than in *ifc-2(kc15)* (Fig. 8b) arguing for impaired nutrient uptake of the intestine. Dietary restriction is known to increase life span^30^. In good agreement with this, the Kaplan-Maier survival curve in Fig. 8c shows that the IFB-2 mutants live longer than the wild type. However, IFC-2 mutants show a significantly reduced life span expectancy, which might be due to the observed excretory canal defects.

### Depletion of intestinal intermediate filaments modulates the response to microbial, oxidative and osmotic stress

It has been suggested that IFs are involved in different types of stress response^31^. We therefore examined, whether and how the absence of either IFB-2 or IFC-2 affects the resilience of worms in different stress paradigms.

To test osmotic stress resistance, which is considered to be one of the most important epithelial properties^32,33^, worms were exposed to 300 mM sodium chloride for 24 h followed by an additional 24 h chase period. As expected the absence of IFB-2 or IFC-2 led to increased lethality compared to the wild type and also in comparison to *ifp-1(kc18)* (N2: 2.72%, 5/184; *ifb-2(kc14)*: 16.55%, 24/145; *ifc-2(kc15)*: 25.96%, 27/104; *ifp-1(kc18)*: 3.75%, 3/80; Fig. 8d). In order to discriminate between alterations caused by IFC-2-dependent intestinal and excretory canal dysfunctions we analyzed an *ifb-1(ok2227)* deletion allele. Since IFB-1 is absent in the intestine but present in the excretory canal^34^, *ok2227* should exclusively report the excretory canal-dependent phenotype. *ifb-1(ok2227)* mutants showed a significantly increased lethality in comparison to the wild type which was, however, less than that observed in *ifc-2(kc15)* (*ifb-1(ok2227)*: 10.00%, 8/80; Fig. 8d). This result clearly shows that IFC-2 depletion affects the organism not only through excretory canal- but also through intestine-dependent functions.

To test for oxidative stress resistance, animals were exposed to the superoxide-inducing chemical paraquat. Loss of either IFB-2 or IFC-2 was associated with increased sensitivity to oxidative stress leading to premature death in comparison to either the wild type, *ifp-1(kc18)* or *ifb-1(ok2227)* (N2: 10 h, n = 176; *ifb-2(kc14)*: 9 h, n = 136; *ifc-2(kc15)*: 6 h, n = 82; *ifp-1(kc18)*: 10 h, n = 77; *ifb-1(ok2227)*: 10 h, n = 79; Fig. 8e).

It has been reported that a dysfunctional endotube is the cause of increased susceptibility to microbial stress^16^. We therefore wanted to explore how loss of IFB-2 and IFC-2 interfere with microbial stress resilience. We observed a significantly increased larval arrest rate in *ifb-2(kc14)* growing on Cry5B producing bacteria compared to *ifc-2(kc15)* and wild-type N2 (Fig. 8f). Time of development was increased in *ifb-2(kc14)* and *ifc-2(kc15)*, although the retardation was most pronounced in *ifb-2(kc14)* (Fig. 8g). Changes in life span were more complex. IFB-2 mutants lived longer on control bacteria than the IFC-2 mutants and even in comparison to the wild type (see Fig. 8c,h). However, application of Cry5B had the strongest effect on the life span reduction of IFB-2 mutants and less on IFC-2 and the wild type (Fig. 8h).

## Discussion

Among intermediate filaments those of epithelial cells are the most diverse with 58 intermediate filament polypeptide-encoding genes in human that are expressed in cell type-, context- and function-dependent patterns^35^. To which degree this astounding diversity reflects redundancy or isotype-specific functions of the different intermediate filament polypeptides has not been fully resolved. Loss of individual polypeptides is of surprisingly little consequence with isotype-specific phenotypes^36^. Complete depletion of the entire type II keratin intermediate filament gene cluster in mice, however, leads to embryonic lethality at E9.5^37^. Remarkably, the production of a single type I keratin, i.e. keratin 18, is sufficient to support complete embryonic development until birth^38^.

The overlapping and differential expression patterns of keratins in the human intestine also suggest a combination of isotype-specific and more general overarching functions (review in^8^). So far, knockout experiments of intestinal keratins have provided limited insight resulting in no detectable intestinal phenotype for K7 and K19^39,40^, keratin network abnormalities without intestinal dysfunction for K18^41^ and mild polarity defects and colonic hyperplasia with colitis for K8^42–45^.

The current study together with previous reports (e.g.^9,11,17–20^) provides conclusive evidence that the six intestinal intermediate filament polypeptides of *C. elegans* are uniformly co-expressed throughout the entire intestinal tube with no major heterogeneities or anterior-posterior gradients. Even more, all polypeptides localize to the same subcellular structure, i.e. the subapical endotube. These findings point to an evolutionary advantage of intestinal intermediate filament diversity, which is likely not based on an essential contribution of individual IFs to intestinal function but rather on adaptive benefits of the changeable IF complement. Thus, a given amount and admixture of IFs may help worms to cope with specific environmental challenges. In agreement, we and others have recently observed an increase in IFB-2, IFC-2 and IFD-2 in response to microbial toxin stress (^16^; see also^46^). Each IF polypeptide may confer slightly different biomechanical and biochemical properties onto the endotube. The unique positioning of the endotube at the interface of two separate subcellular compartments likely translates these modifications into functional alterations. For example, isotype-specific tuning of endotube elasticity may influence the recoil of the stiff apical brush border thereby contributing to lumen maintenance and, most importantly, to efficient food processing and nutrient uptake. This notion is supported by the observed luminal widening in *ifo-1*, *ifb-2* and *ifc-2* mutants and the local invaginations in *sma-5* mutants presenting a discontinuous endotube leading to reduced body size and progeny (this study and^12,15^). Recent studies on vimentin IFs in mammalian cells further underline the important mechanophysical function of IFs in living cells by demonstrating that the hyperelastic network contributes to maintenance of cell viability at large deformations^47^.

The comparatively mild functional impairments in the *ifb-2* null allele *kc14* compared to that observed in *ifo-1* and *sma-5* mutants (Supplementary Table S2) suggest negative effects of the junctional IF aggregates in *ifo-1* mutants and of the endotube irregularities in *sma-5* mutants. In addition, IF-independent functions of IFO-1 and SMA-5 may also be the reason for the more severe phenotypes. Comparing the phenotypes of the different IF mutant alleles identifies IFB-2 as the master endotube organizer with detectable but less contribution by IFD-2 and IFC-2 and none by the other investigated IFs. The most likely explanation is that IFB-2 pairs with either of the other intestinal IF polypeptides, which may only have a limited or no capacity to form filaments on their own. This view is supported by overlay assays of recombinant polypeptides^48^. They show that IFB-2 binds to IFC-1, IFC-2, IFD-1 and IFD-2, although homo- and heterophilic binding also occurs among the other polypeptides. Overlay assays, however, do not allow to distinguish between different binding strengths and do not provide direct information on filament-forming capacity. Another explanation of the preeminent function of IFB-2 in endotube formation maybe the abundance of this polypeptide. Immunoblot comparison of the expression level of the IFB-2 reporter (which is expressed at comparable level to the endogenous IFB-2) and the IFC-2 reporter (Supplementary Fig. S2) showed that IFB-2 is at least an order of magnitude more abundant than IFC-2. A special situation is encountered for IFD-2. It is not only highly responsive in different stress paradigms (see^16,46^) but also presents with a unique phenotype, i.e. intestinal luminal widening with cytoplasmic invaginations. More detailed analyses on the precise distribution of IFD-2 in the intestine as well as ultrastructural and functional analyses of IFD-2-depleted worms are needed to further clarify its specific properties.

The consequences of deleting intestinal IFs for other structural abnormalities in the intestine, for development, life span and stress resilience do not follow a simple rule. This may be due to additional isotype-specific functions both within the intestine and other sites, the full elucidation of which requires additional experiments. The comparison of IF mutants in this report (see also summary in Supplementary Table S2), however, has provided information on

- *shared intestinal functions:* IFB-2 and IFC-2 affect intestinal properties albeit to different degrees. Thus, more severe alterations are detected for loss of IFB-2 concerning endotube thickness, luminal widening and microvillar disorder resulting in developmental retardation, brood size reduction and in larval arrest in response to Cry5B intoxication.
- *redundant intestinal functions:* Redundant functions may be fulfilled by IFC-1 and IFP-1 with respect to endotube formation. In accordance, the acute osmotic stress response of *ifp-1(kc18)* was not compromised. More sensitive assays are therefore needed to evaluate the relative contribution of these polypeptides for intestinal morphology and function. The same may apply to IFC-1 and IFD-1 both of which are expressed in the intestine and, based on RNAi experiments, also fulfil redundant functions^18,49^.
- *isotype-specific intestinal functions*: The differential response of actin and the actin-bundling protein PLST-1 to loss of IFB-2 and IFC-2 marks such a situation. It is in line with the keratin 19-specific plastin-association in murine intestine, which was shown to mediate microvillar actin rootlet anchorage^28^. The unexpected increased life span of *ifb-2(kc14)* is another example of isotype specificity caused by unique properties of IFB-2, the nature of which still remains to be elucidated. A possibility is that it is related to the observed reduced brood size.
- *non-intestinal functions:* This is the case for IFC-2, which is produced in the excretory canal. This non-intestinal expression is responsible for the increased acute osmotic and oxidative stress sensitivity of *ifc-2(kc15)* compared to *ifb-2(kc14)*.

With the IF mutants we have a useful toolbox in hand for further quantitative analyses. It is our firm belief that only such assessments, which are almost impossible to perform in transgenic mice, are essential to understand the modulatory function of IF polypeptides *in vivo* at steady state and, even more importantly, in defined stress situations. By systematically analyzing single mutants and various combinations thereof, we will be able to measure graded responses to mechanophysical, chemical or microbial challenges that typically confront epithelial tissues *in situ*.

## Methods

### DNA constructs

To prepare constructs for CRISPR-mediated knockouts, *ifc-2*- or *ifp-1*-specific sgRNAs were inserted into plasmid pDD162 (Peft-3::cas9 + empty sgRNA, Addgene, Cambridge, MA, #47549) by rolling circle mutagenesis PCR using the Q5^®^ Site-Directed Mutagenesis Kit (NEB, Frankfurt, Germany). Primers 16-093 (GGCTTTGGCCAGCTTGTCAGGTTTTAGAGCTAGAAATAGCAAGT) and 15-010 (CAAGACATCTCGCAATAGG) were used for *ifc-2* and 17-023 (CATGGATTCCGCTAACGCTAGTTTTAGAGCTAGAAATAGCAAGT) and 15-010 for *ifp-1* generating plasmid constructs #4123 and #4135, respectively. *ifc-1p::ifc-1::egfp;unc-119*(+) containing fosmid clone 18076040888727174 F05, *ifd-2p::ifd-2::egfp;unc-119*(+) containing fosmid clone 095794875301489 H04, and *ifp-1p::ifp-1::egfp;unc-119*(+) containing fosmid clone 23762707929976346 G11 were obtained from the Transgeneome Project, MPI Dresden, Germany (transgeneome.mpi-cbg.de for sequence details^50^). Plasmid pCFJ104 [*myo-3p::mCherry::unc-54*] was used as selection plasmid for microinjection. For RNAi experiments clones of the Ahringer feeding library (Source BioScience, Nottingham, UK) were used.

### *C. elegans* strains and bacteria

A summary of all strains used in this study is shown in Supplementary Table S1.

The following DNA concentrations (dissolved in ddH_2_O) were used for microinjection. Fosmid clones 18076040888727174 F05, 095794875301489 H04 and 23762707929976346 G11: 40 ng/µl; knockout plasmids #4113, #4123 and #4135: 40 ng/µl; selection plasmid pCFJ104: 80 ng/µl; carrier DNA: 80 ng/µl.

Integration of extrachromosomal fosmids was done by using X-ray radiation. To this end, 10 L4 larvae were transferred on a OP50-containing NGM agar plate and were exposed to 4000 rad. They were then grown until starvation. A 2 cm × 2 cm agar piece was chunked from each plate to a new one. Worms were grown for another generation followed by cloning 20 animals from each plate onto a new plate. Plates with 100% array transmission in their progeny were considered to be stable integrants. Homozygous strains were then outcrossed with N2.

OP50 bacteria were obtained from the CGC. JM103 *E. coli* carrying either the pQE9 control vector (referred to as control bacteria) or the pQE9 vector containing the Cry5B gene (referred to as Cry5B-producing bacteria) were kindly provided by Raffi Aroian^51^.

RNAi by feeding was performed as previously described^12^ without supplement of tetracycline.

### Intoxication assays

The Cry5B intoxication experiments were performed as described^16^. In brief, empty pQE9 vector-containing JM103 or Cry5B-producing JM103 were grown over night in Luria broth (LB) with 100 µg/ml ampicillin and 7 µl/ml 1 M isopropyl β-d-1-thiogalactopyranoside (IPTG). 300 µl aliquots were placed on nematode growth medium (NGM) plates containing 100 µg/ml ampicillin and 2 mM IPTG. Inoculated plates were incubated overnight at room temperature and plates were stored at 4 °C. Intoxication experiments were started by transferring adult worms into 70 µl bleaching solution containing 1 ml PBS, 150 µl 13% NaOCl and 100 µl 4 M NaOH next to the bacterial lawn. Mother animals got dissolved thereby releasing their viable embryos. Plates were incubated at 18 °C during the entire experiment. Animals were transferred at least every three days onto new plates.

### Stress assays

Stress assays were performed as described^16^. For oxidative stress we used 200 mM methyl viologen dichloride hydrate (paraquat; Sigma-Aldrich, Munich, Germany) in NGM agar. Poured plates were stored overnight at room temperature and subsequently inoculated with 50 µl 10 x concentrated OP50 overnight culture. Following two days of room temperature incubation plates were stored for a maximum of three days at 4 °C until further usage. Oxidative stress experiments were started by transferring L4 larvae onto the bacterial lawn and incubation at 18 °C. Animals were hourly scored for viability using mechanical stimulation with a platinum wire. No response animals were scored dead. Plates without paraquat were used as controls. Statistical analysis was done using the survival function and the Gehan-Breslow-Wilcoxon Test of GraphPad Prism 5.01 (GraphPad Software Inc., LaJolla, CA).

For osmotic stress we used NGM agar with 300 mM NaCl. Poured plates were stored overnight at room temperature and subsequently inoculated with 300 µl OP50 overnight culture. Following overnight incubation at room temperature plates were ready to use. Osmotic stress experiments were started by transferring L4 larvae onto the bacterial lawn followed by an overnight incubation at 18 °C. Subsequently, worms were washed in recovery buffer (M9 buffer with 150 mM NaCl) and transferred to normal NGM plates. The viability of each animal was scored after an additional overnight incubation at 18 °C using mechanical stimulation through a platinum wire. Data are represented as mean ± SD. The Chi square function of Excel (Microsoft, Redmont, WA) was used for significance calculation.

### Analysis of larval development and progeny

Isolated embryos were placed onto each plate, immediately counted and incubated at 18 °C. Individual larvae were monitored every day for animals becoming adult during their entire life span. Adults were counted and removed from the plates to prevent double-counting. The remaining larvae were transferred at least every three days to prevent mix-up with progeny animals. Larval arrest rates in % were calculated from the ratio of animals dying as larvae and the total number of counted larvae on the plate. Data were represented as mean ± SD. Significance was determined by using the Chi-square function of Excel. Time of development was calculated by the average number of days, animals needed to reach adulthood. Progeny of these animals was counted during their entire life span and is given as an average number per individual. Data were represented as mean ± SD. For significance the unpaired, two-tailed t-test function of GraphPad Prism 5.01 was used.

### Life span analysis

Embryos were isolated and incubated at 18 °C. Subsequently hatched animals where scored for viability every day using a platinum wire for mechanical stimulation. Animals moving upon stimulation were judged to be alive. Animals that could not be located on the plate throughout the experiments were excluded. Worms were transferred at least every three days to prevent mix-up with their hatched progeny. Survival data were compiled as a plots of the Kaplan–Meier estimator. Statistical analysis was performed using the survival function and the Gehan-Breslow-Wilcoxon Test of GraphPad Prism 5.01.

### Light and Brillouin microscopy

For light microscopy a confocal laser-scanning microscope (LSM710, Zeiss Jena, Germany) and a Zeiss Apotome with a Zeiss AxioCamMRm were used.

Brillouin microscopy is a purely optical technique that allows acquiring 3D maps of viscoelastic properties with diffraction limited resolution^25,52^. It is based on Brillouin scattering, the interaction of monochromatic laser light with thermally excited, spontaneous sound waves in the GHz frequency range that exists in all matter. Due to this interaction, a small amount of the laser light exchanges energy with the sound waves and thus obtain a change in frequency. This change in frequency, termed ‘Brillouin shift’, is given by:

$${\nu }_{B}=\frac{2n}{{\lambda }_{0}}V\,\sin \,\frac{\theta }{2}$$

where $n$ is the refractive index within the interaction volume, ${\lambda }_{0}$ is the wavelength of the laser in vacuum, $\theta$ is the angle between incident and scattered light and $V$ the speed of sound. Importantly, the speed of sound $V$ is related to the real part of the longitudinal modulus $M\text{'}$, a measure of ‘stiffness’, and is defined as the ratio of stress to strain in a uniaxial strain state:

$$M{\prime} =\rho {V}^{2}$$

To determine the elastic modulus $\,M\text{'}$, the knowledge of the refractive index $n$ and the density $\rho$ is required. However, it was shown that in most biological samples $n$ and $\rho$ are correlated to within an error of few percent, thus not affecting the value of $M\text{'}$^53^. Therefore, in the absence of *in-situ* measures of $n$ and $\rho$ we report the Brillouin shift as the proxy for stiffness as it is the quantity measured in our experiments. Regions within an image displaying a higher Brillouin shifts are thus relatively ‘stiffer’.

For Brillouin imaging worms were mounted on 2% agar pads anaesthetized with 10% levamisole. An 18 × 18 mm coverslip was placed on top and petroleum jelly was used for sealing. Brillouin images were acquired using a custom-built confocal Brillouin microscope based on a commercial Zeiss body (Axiovert 200) coupled with a 532 nm laser and a custom-built 2-VIPA spectrometer, as described in more detail previously^24^. Furthermore, the setup allows to perform sequential confocal fluorescence imaging, using 532 nm or 488 nm as excitation wavelengths.

The intensity of the laser light was adjusted to <5 mW on the sample to avoid photodamage. The light was focused using 40 × 1.0 NA oil objective. The images were acquired by scanning the sample with a step size of 0.5 µm and an acquisition time of 180 ms per pixel for Fig. 4A and 0.3 µm step size and 210 ms acquisition time per pixel for Fig. 4B. Fluorescence confocal images were acquired immediately before Brillouin images with a step size of 0.25 µm.

### Fluorescence staining with labeled antibodies, phalloidin and dextran

For immunostaining, animals were decapitated in 1x PBS on a poly-l-lysine-coated glass slide followed by mounting a coverslip on top, removing excessive liquid and freezing the sample in liquid nitrogen. Forceful removal of the coverslip with a scalpel resulted in permeabilization of the cuticle. Samples were processed as follows: 5-10 min in methanol, 10–20 min in acetone (both steps at −20 °C), followed by incubation in a graded ethanol series (5 min each in 90% / 60% ethanol at −20 °C and 5 min in 30% ethanol at room temperature) and 10 min washing in TBST (TBS buffer [20 mM (w/v) tris(hydroxymethyl)-aminomethane pH 7.6, 0.15 M (w/v) NaCl] + 0.2% Tween 20). Subsequently, samples were incubated with primary antibodies dissolved in blocking solution (1% (w/v) non-fat milk powder (Roth, Karlsruhe, Germany), 1% (w/v) bovine serum albumin and 0.02% (w/v) sodium azide) overnight at 4 °C. After a 10 min washing step in TBST samples were incubated with secondary antibodies dissolved in blocking solution for 2 h at room temperature. After washing for 10 min in TBST samples were embedded in Mowiol 4–88 (Sigma-Aldrich, Hamburg, Germany) supplemented with DABCO (Roth) and covered with a glass coverslip.

The following primary antibodies were used: Mouse monoclonal anti-IFB-2 antibody MH33 (1:100, Developmental Studies Hybridoma Bank^54^), anti-ERM-1 antibody ERM1 (1:200, Developmental Studies Hybridoma Bank^54^) and rabbit polyclonal anti-GFP antibody (1:1000, Invitrogen, Carlsbad, CA, #A-11122).

In addition, an anti-IFC-2 antibody was prepared in guinea pig using recombinant IFC-2e. For the expression of IFC-2e in *E. coli* bacteria, we cloned the coding sequence of *ifc-2e* into the NheI/BamHI sites of plasmid pET24a(+) (Merck, Darmstadt, Germany, #69749) giving rise to plasmid pIFC-2e (#1856). The isolation of inclusion bodies and column purification were performed according to^55^. Peptide Specialty Laboratories (Heidelberg, Germany) performed immunization of two guinea pigs using 1 mg of purified IFC-2e protein. Injections were conducted every two weeks with a total of 4 per animal followed by bleeding. Specificity and optimal dilution (1:100) of antibody was tested by immunofluorescence using wild-type and *ifc-2(kc15)* animals (Fig. 6l–m˝). Specific signals in immunoblots were not obtained.

Secondary antibodies were Alexa Fluor 488-conjugated affinity-purified anti-mouse IgG (1:200, Invitrogen, Carlsbad, CA, #A-11029), Alexa Fluor 555-conjugated highly cross-absorbed anti-mouse IgG (1:200, Invitrogen, Carlsbad, CA, #A-21424), Alexa Fluor 647-conjugated affinity-purified anti-mouse IgG (1:200, Invitrogen, Carlsbad, CA, #A-21236), Alexa Fluor 488-conjugated affinity-purified anti-rabbit IgG (1:200, Invitrogen, Carlsbad, CA, #A-11070), Cy3-conjugated anti-guinea pig IgG (1:200, Jackson/Dianova, Cambridgeshire, United Kingdom, #93443) and Dylight 488-conjugated anti-guinea pig IgG (1:200, Jackson/Dianova, Cambridgeshire, United Kingdom, #95746).

Formaldehyde fixation was used for phalloidin staining. After freeze crack, samples were incubated with fixation solution (75% methanol, 3,7% formaldehyde, 4% Alexa Fluor 488-conjugated phalloidin (Molecular Probes, Eugene, OR, #A-12379), 11% dH_2_O) for 30 min at −20 °C. The following steps were performed as described above with additional use of phalloidin dissolved 1:25 together with the primary antibodies.

For co-localization analysis (Fig. 2) intestines were isolated, fixed and stained as described above. Imaging was performed using a Zeiss LSM710 with Airyscan. Fiji was used for distance quantification using the mean of 100 fluorescence profiles taken across the intestinal lumen covering the boxed area in d.

For quantification of stainings intestines were isolated, imaged with a Zeiss Apotome microscope (Fig. 7) using identical settings in a single session. Resulting stacks were further processed using the sum-slice projection function of Fiji (https://fiji.sc/). For *in vivo* quantification single plane images were used. Regions of interest were manually defined. The average greyscale intensity per pixel was measured using the measurement function of Fiji. Significance was evaluated using the unpaired, two-tailed t-test function of GraphPad Prism 5.01 (GraphPad Software, Inc., LaJolla, CA).

For fluorescence labelling of the intestinal lumen age staged animals were transferred to plates containing 10.000 MW Alexa Fluor 546 labelled dextran (Molecular Probes, Eugene, OR, D-22911) in the presence of OP50 bacteria and incubated at 18 °C. Prior to imaging worms were thoroughly washed in M9 buffer. Fluorescence was subsequently recorded and scored for individuals showing luminal dilation.

### Electron microscopy

High-pressure cryofixation was used for electron microscopy^16^ by transferring young adult worms into a 100 µm deep membrane carrier prefilled with 20% BSA in M9 worm buffer (22 mM KH_2_PO_4_, 42 mM Na_2_HPO_4_, 86 mM NaCl, 1 mM MgSO_4_) and high pressure freezing using a Leica EM Pact high-pressure freezer (Leica, Wetzlar, Germany). A minimum of 5 samples consisting of 10–20 worms were fixed per experiment. Consecutively quick freeze substitution was performed using 1% OsO_4_, 0.2% uranyl acetate in acetone followed by epoxy resin embedding as previously described^56^. 50 nm sections were prepared using a Leica UC6/FC6 ultramicrotome, contrasted for 10 min each in 1% uranyl acetate in ethanol and Reynolds lead citrate and finally imaged at 100 kV using a Hitachi H-7600 transmission electron microscope (Tokyo, Japan).

Endotube thickness was measured in areas with longitudinally sectioned microvilli using the measurement function of Fiji.

For immunogold labelling high-pressure frozen worms were rapidly freeze substituted with the help of a Leica AFS fitted with an agitation module^57^ in 0.2% uranyl acetate, 5% H_2_O in acetone as follows: from −110 °C up to −50 °C over 2 hours, pause at −50 °C, 2 × 5 min washes with acetone, 2 × 5 min washes with ethanol, successive 20%, 40%, 60%, 80%, and 100% Lowicryl (Electron Microscopy Sciences, Hatfield, PA) over 2 hours and overnight incubation in 100% Lowicryl. Polymerization took place over the next 24 hours during slow warming to room temperature. After polymerization, 40–50 nm sections were collected onto Formvar-coated nickel grids. Sections were incubated for 20 min at room temperature in 0.1% glycine in PBS then blocked for 30 min in 1% (w/v) BSA in PBS. The sections were incubated for 30 min in primary antibody diluted 1 in 20 with 0.1% BSA in PBS. After three 5 min washes in 1% BSA in PBS they were incubated with 5 nm gold secondary antibody (British Biocell International, Cardiff, UK) diluted 1:20 in 1% BSA in PBS for 30 min. For negative controls primary antibody was omitted. After washing with distilled water, sections were either stained with Reynolds lead citrate for 5 min or sometimes staining was omitted to help improve the visibility of the small 5 nm gold particles on some tissues. Sections were imaged with a Hitachi H-7600 transmission electron microscope at 100 kV.

### Immunoblotting

For each assay 60 larvae of wild-type or *plst-1(tm4255)* animals were handpicked, transferred to 30 µl dH2O and immediately frozen at −80 °C. Samples were quickly thawed and sucked up and down three times through a 30 G hypodermic syringe (BD Medical, Heidelberg, Germany). Subsequently, 7.5 µl of 5x Laemmli loading buffer was added followed by incubation for 5 min at 90 °C. Protein separation was performed using a 10% sodium dodecyl sulfate (SDS) polyacrylamide gel. Proteins were then transferred onto a polyvinylidene difluoride (PVDF) membrane by wet tank-blotting at 100 V for 60 min. Membranes were blocked for 2 hours at room temperature using Roti®-Block (Roth) followed by primary antibody incubation in Roti®-Block at 4 °C overnight. The following primary antibody dilution were used: Mouse monoclonal anti-IFB-2 antibody MH33 (1:1000 dilution in Roti®-Block) and rabbit polyclonal anti-actin antibody (1:1000 dilution in Roti®-Block, Sigma-Aldrich, #A2066).

For cytoskeletal protein extraction, three 10 cm NGM plates with many adult animals were pelleted in M9 buffer, transferred into a precooled mortar filled with liquid nitrogen and ground to a fine powder. Subsequently, 6 ml lysis buffer (50 mM KCl, 10 mM Tris-HCl (pH 8.3), 2.5 mM MgCl2, 0.45% (v/v) nonident P-40, 0.45% (v/v) Tween-20, 0,01% (w/v) gelatine, supplemented with Roche complete protease inhibitor cocktail) was added and transferred to three 2 ml Eppendorf tubes. After 15 min agitation at 8 °C followed by 15 min centrifugation at 20800 × g and 4 °C, the supernatant was collected in a 15 ml Falcon tube and protein content was measured using the DC^TM^ Protein Assay (Biorad, Feldkirchen, Germany). Each pellet was washed with 1 ml lysis buffer followed by a second centrifugation step. After removal of the lysis buffer pellets were resuspended in 40 µl 2x Laemmli loading buffer and collected in a single Eppendorf tube followed by incubation for 5 min at 100 °C and storage on ice. Another centrifugation step was applied to remove debris. A volume of 30 or 22.5 µl per lane (to balance estimated protein levels) was loaded onto a 10% SDS polyacrylamide gel. The separated proteins were subsequently blotted on a PVDF membrane, which was finally blocked for 1 h using 5% milk powder (Roth) dissolved in TBST (blocking solution). Primary antibody incubation was carried out using rabbit polyclonal anti-GFP (1:1000 in blocking solution, abcam, Berlin, Germany, #ab183734) overnight at 4 °C.

Three times washing with TBST (20 mM tris(hydroxymethyl)-aminomethane, 0.15 M NaCl, 0.1% Tween 20 (v/v), pH 7.6) was performed before incubation with secondary antibodies (goat anti-mouse IgG antibodies and goat anti-rabbit IgG antibodies coupled to horseradish peroxidase from DAKO at 1:5000 in Roti®-Block) for 1 h at room temperature followed by washes (3 × 10 min) in TBST. Bound antibodies were detected by chemiluminescence substrate AceGlow (VWR, Darmstadt, Germany, #730-1510) using a Fusion Solo (Vilber Lourmat, Eberhardzell, Germany).

## Supplementary information

Supplementary Information.

## Data Availability

All data generated or analyzed during this study are included in this article and its Supplementary Information Files.

## References

[1] Baumgart DC, Dignass AU (2002). Intestinal barrier function. Curr. Opin. Clin. Nutr. Metab. Care.

[2] Chelakkot C, Ghim J, Ryu SH (2018). Mechanisms regulating intestinal barrier integrity and its pathological implications. Exp. Mol. Med..

[3] Crawley SW, Mooseker MS, Tyska MJ (2014). Shaping the intestinal brush border. J. Cell Biol..

[4] Turner JR (2009). Intestinal mucosal barrier function in health and disease. Nat. Rev. Immunol..

[5] Bement, W. M. & Mooseker, M. S. The cytoskeleton of the intestinal epithelium: Components, assembly, and dynamic rearrangements. In The Cytoskeleton: A Multi-Volume Treatise, Vol. 3 (ed. John, E. H. and Ian, F. P.). *JAI Press*, *Greenwich*, 359–404 (1996).

[6] Hirokawa N, Tilney LG, Fujiwara K, Heuser JE (1982). Organization of actin, myosin, and intermediate filaments in the brush border of intestinal epithelial cells. J. Cell Biol..

[7] Brunser O, Luft HJ (1970). Fine structure of the apex of absorptive cell from rat small intestine. J. ultrastructure Res..

[8] Coch Richard, Leube Rudolf (2016). Intermediate Filaments and Polarization in the Intestinal Epithelium. Cells.

[9] Bossinger O, Fukushige T, Claeys M, Borgonie G, McGhee JD (2004). The apical disposition of the Caenorhabditis elegans intestinal terminal web is maintained by LET-413. Dev. Biol..

[10] Carberry K, Wiesenfahrt T, Windoffer R, Bossinger O, Leube RE (2009). Intermediate filaments in Caenorhabditis elegans. Cell Motil. Cytoskeleton.

[11] Husken K (2008). Maintenance of the intestinal tube in Caenorhabditis elegans: the role of the intermediate filament protein IFC-2. Differ..

[12] Geisler F (2016). A novel function for the MAP kinase SMA-5 in intestinal tube stability. Mol. Biol. Cell.

[13] Jahnel O, Hoffmann B, Merkel R, Bossinger O, Leube RE (2016). Mechanical Probing of the Intermediate Filament-Rich Caenorhabditis Elegans Intestine. Methods Enzymol..

[14] Block J, Schroeder V, Pawelzyk P, Willenbacher N, Koster S (2015). Physical properties of cytoplasmic intermediate filaments. Biochim. Biophys. Acta.

[15] Carberry K (2012). The novel intestinal filament organizer IFO-1 contributes to epithelial integrity in concert with ERM-1 and DLG-1. Dev..

[16] Geisler Florian, Coch Richard A., Richardson Christine, Goldberg Martin, Denecke Bernd, Bossinger Olaf, Leube Rudolf E. (2019). The intestinal intermediate filament network responds to and protects against microbial insults and toxins. Development.

[17] Karabinos A, Schulze E, Klisch T, Wang J, Weber K (2002). Expression profiles of the essential intermediate filament (IF) protein A2 and the IF protein C2 in the nematode Caenorhabditis elegans. Mech. Dev..

[18] Karabinos A, Schunemann J, Weber K (2004). Most genes encoding cytoplasmic intermediate filament (IF) proteins of the nematode Caenorhabditis elegans are required in late embryogenesis. Eur. J. Cell Biol..

[19] Karabinos A, Schulze E, Schunemann J, Parry DA, Weber K (2003). *In vivo* and *in vitro* evidence that the four essential intermediate filament (IF) proteins A1, A2, A3 and B1 of the nematode Caenorhabditis elegans form an obligate heteropolymeric IF system. J. Mol. Biol..

[20] Karabinos A, Schulze E, Baumeister R (2019). Analysis of the novel excretory cell expressed ECP-1 protein and its proposed ECP-1/IFC-2 fusion protein EXC-2 in the nematode Caenorhabditis elegans. Gene Expr. Patterns.

[21] Al-Hashimi H, Hall DH, Ackley BD, Lundquist EA, Buechner M (2018). Tubular Excretory Canal Structure Depends on Intermediate Filaments EXC-2 and IFA-4 in Caenorhabditis elegans. Genet..

[22] Khan LA (2019). A tensile trilayered cytoskeletal endotube drives capillary-like lumenogenesis. J. Cell Biol..

[23] Dodemont H, Riemer D, Ledger N, Weber K (1994). Eight genes and alternative RNA processing pathways generate an unexpectedly large diversity of cytoplasmic intermediate filament proteins in the nematode Caenorhabditis elegans. EMBO J..

[24] Bevilacqua C, Sanchez-Iranzo H, Richter D, Diz-Munoz A, Prevedel R (2019). Imaging mechanical properties of sub-micron ECM in live zebrafish using Brillouin microscopy. Biomed. Opt. Express.

[25] Prevedel Robert, Diz-Muñoz Alba, Ruocco Giancarlo, Antonacci Giuseppe (2019). Brillouin microscopy: an emerging tool for mechanobiology. Nature Methods.

[26] Scarcelli G (2015). Noncontact three-dimensional mapping of intracellular hydromechanical properties by Brillouin microscopy. Nat. Methods.

[27] Claessens MMAE, Bathe M, Frey E, Bausch AR (2006). Actin-binding proteins sensitively mediate F-actin bundle stiffness. Nat. Mater..

[28] Grimm-Gunter EM (2009). Plastin 1 binds to keratin and is required for terminal web assembly in the intestinal epithelium. Mol. Biol. Cell.

[29] Ding WY (2017). Plastin increases cortical connectivity to facilitate robust polarization and timely cytokinesis. J. Cell Biol..

[30] Piper MD, Bartke A (2008). Diet and aging. Cell Metab..

[31] Toivola DM, Strnad P, Habtezion A, Omary MB (2010). Intermediate filaments take the heat as stress proteins. Trends Cell Biol..

[32] D’Alessandro M, Russell D, Morley SM, Davies AM, Lane EB (2002). Keratin mutations of epidermolysis bullosa simplex alter the kinetics of stress response to osmotic shock. J. Cell Sci..

[33] Pekny M, Lane EB (2007). Intermediate filaments and stress. Exp. Cell Res..

[34] Williams K, Williams K, Baucher HM, Plenefisch J (2015). The tail domain is essential but the head domain dispensable for C. elegans intermediate filament IFA-2 function. PLoS One.

[35] Moll R, Divo M, Langbein L (2008). The human keratins: biology and pathology. Histochem. Cell Biol..

[36] Bouameur JE, Magin TM (2017). Lessons from Animal Models of Cytoplasmic Intermediate Filament Proteins. Subcell. Biochem..

[37] Vijayaraj P (2009). Keratins regulate protein biosynthesis through localization of GLUT1 and -3 upstream of AMP kinase and Raptor. J. Cell Biol..

[38] Kumar V (2015). A keratin scaffold regulates epidermal barrier formation, mitochondrial lipid composition, and activity. J. Cell Biol..

[39] Sandilands A (2013). Generation and characterisation of keratin 7 (K7) knockout mice. PLoS One.

[40] Tamai Y (2000). Cytokeratins 8 and 19 in the mouse placental development. J. Cell Biol..

[41] Magin TM (1998). Lessons from keratin 18 knockout mice: formation of novel keratin filaments, secondary loss of keratin 7 and accumulation of liver-specific keratin 8-positive aggregates. J. Cell Biol..

[42] Baribault H, Penner J, Iozzo RV, Wilson-Heiner M (1994). Colorectal hyperplasia and inflammation in keratin 8-deficient FVB/N mice. Genes. Dev..

[43] Ameen NA, Figueroa Y, Salas PJ (2001). Anomalous apical plasma membrane phenotype in CK8-deficient mice indicates a novel role for intermediate filaments in the polarization of simple epithelia. J. Cell Sci..

[44] Toivola DM, Krishnan S, Binder HJ, Singh SK, Omary MB (2004). Keratins modulate colonocyte electrolyte transport via protein mistargeting. J. Cell Biol..

[45] Asghar MN (2015). The amount of keratins matters for stress protection of the colonic epithelium. PLoS One.

[46] Treitz C, Cassidy L, Hockendorf A, Leippe M, Tholey A (2015). Quantitative proteome analysis of Caenorhabditis elegans upon exposure to nematicidal Bacillus thuringiensis. J. Proteom..

[47] Hu J (2019). High stretchability, strength, and toughness of living cells enabled by hyperelastic vimentin intermediate filaments. Proc. Natl Acad. Sci. USA.

[48] Karabinos A, Schunemann J, Parry DA (2017). Assembly studies of six intestinal intermediate filament (IF) proteins B2, C1, C2, D1, D2, and E1 in the nematode C. elegans. Cytoskeleton.

[49] Karabinos A, Schmidt H, Harborth J, Schnabel R, Weber K (2001). Essential roles for four cytoplasmic intermediate filament proteins in Caenorhabditis elegans development. Proc. Natl Acad. Sci. USA.

[50] Sarov M (2012). A genome-scale resource for *in vivo* tag-based protein function exploration in C. elegans. Cell.

[51] Bischof LJ, Huffman DL, Aroian RV (2006). Assays for toxicity studies in C. elegans with Bt crystal proteins. Methods Mol. Biol..

[52] Scarcelli G, Yun SH (2007). Confocal Brillouin microscopy for three-dimensional mechanical imaging. Nat. Photonics.

[53] Schlussler R (2018). Mechanical Mapping of Spinal Cord Growth and Repair in Living Zebrafish Larvae by Brillouin Imaging. Biophys. J..

[54] Francis R, Waterston RH (1991). Muscle cell attachment in Caenorhabditis elegans. J. Cell Biol..

[55] Herrmann H, Kreplak L, Aebi U (2004). Isolation, characterization, and *in vitro* assembly of intermediate filaments. Methods Cell Biol..

[56] McDonald KL, Webb RI (2011). Freeze substitution in 3 hours or less. J. Microsc..

[57] Reipert S (2018). Agitation Modules: Flexible Means to Accelerate Automated Freeze Substitution. J. Histochem. Cytochem..

