# Supplementary material for: Intestinal intermediate filament polypeptides in C. elegans: Common and isotype-specific contributions to intestinal ultrastructure and function
Source: Sci Rep. 2020 Feb 21;10:3142. doi: 10.1038/s41598-020-59791-w (PMC7035338; doi:10.1038/s41598-020-59791-w)
Supplement: Supplementary file 1 — Supplementary Information. [file 41598_2020_59791_MOESM1_ESM.pdf]

Supplementary Information

Intestinal intermediate filament polypeptides in *C. elegans*: Common and isotype-specific contributions to intestinal ultrastructure and function

Florian Geisler[1], Richard A. Coch[1], Christine Richardson[2], Martin Goldberg[2], Carlo Bevilacqua[3], Robert Prevedel[4], Rudolf E. Leube[1,*]

[1]Institute of Molecular and Cellular Anatomy, RWTH Aachen University, Aachen, Germany

[2]School of Biological and Biomedical Sciences, Durham University, Durham, United Kingdom

[3]Cell Biology and Biophysics Unit, European Molecular Biology Laboratory, Collaboration for joint PhD degree between EMBL and Heidelberg University, Faculty of Biosciences, Heidelberg, Germany

[4]Cell Biology and Biophysics Unit, European Molecular Biology Laboratory, Heidelberg, Germany

[*]Corresponding author:
Rudolf Leube
Institute of Molecular and Cellular Anatomy
RWTH Aachen University
Wendlingweg 2
52074 Aachen, Germany
Phone: ++49 241 80 89107
Fax: ++49 241 80 82508
Email: rleube@ukaachen.de

a

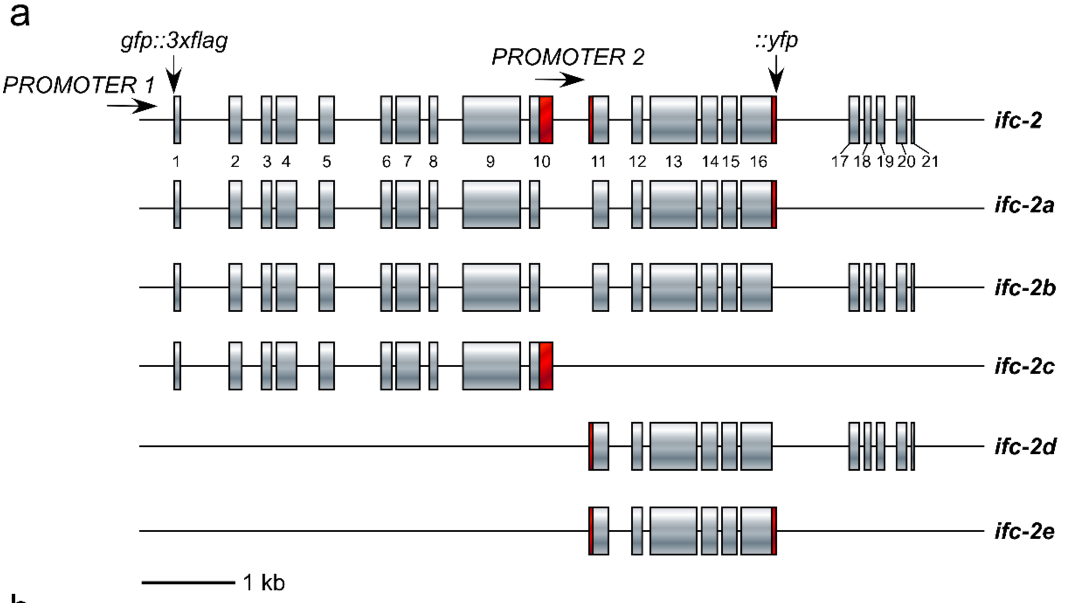

b

IFC-2, isoform a 1128 aa

MSLTSTPFSTDDERYSGYTNSAMSPPLQKQLIQEVSLSYSAPYTSTVKPSRHDLPLAFSPYSLDSEIEDLHSHTYSQLSNRSNGNKVE
SAERIYTASRKETEFPAKADPIPYFSAQSAIKAGRESPSYTTRSTPTPRQTPISFDQASTARLDSGLSQNLIVSESTTFEELTTITTS
NNRTTRKKEIKTEMPTVNALTKQFEISSQQEKVVPLKPRKSSNVDYNGNGNSRSQNGTYTPNRSAIVYSPGGILTTTIVDIEPARFGR
RTEEYKIQEEYIVQRYDNGSSPNYQRQEQGYQQQQQHRSREYKTHRQEKQENYNQRSYSATENREVDAPVDTSPIDVVKNVIDRWETK
IGGTTTRFYASDQQLRSVSRVDMNDNVPRSAQEKIIVHQRSESQPTERVIPISPLPHRVEPTRTTQSGGLLQTYEELKTVVVKQSSDQ
TPQKIVPRAQSPVTSPVFKETILSPPPIPTPSPTSDIAEEVLRTDETTQHTAITMHSIVTKEVKQEAKEKINSVTDNAEMKEKVVEIA
KNVEYEWEKKPLTPTPTPIPPQQQEVKQEELKVENKIENDQNEVHLHKFLDRDYRIKTYEEYSTTKRLRDRSQPPERPPTSYSSYSDR
SRSIDRTYERRVLGSGMSRTYSEGRGLASGTYASGFGQLVSGMSSAGAICTTQIRDAREREKREIGLLNDRLADYIEKVRFLEAQNRC
LSHDIDILRNGFSGGGHVSGLFDAEINQAKHILEQTTAHRSTFERDITGLSAEIEVFRKKWLDAVNAVKAHREDHDVDLDRLAKIEAE
ISLFKRKIRIVEEDVIRIRRENDGIYNEIARIKQLTHNEIALKNERSLNVSDLLQRINLLQTENNVRIEQELVFIRRDTTADNRDYFR
HELQAAIRDIRADYEAISIRNRQDIEVWYREQIRKIQDESVRVNPDLYKEELISIRTTVTNVRSRLAEVEGRNFFLERLIDDLKNNEE
AKFFEISLAERDAQIATLRDQCTELSIQMEKLCDNEISLRAEIERYRILLNGANVTTYTSNTHGSGSGIAVGGVVGGSTRVISQTTRT
HSSSNTSYSNVPASRGGYSISGNVGGISVGGTIGSHGASAHATGGIIGSGVQAHRGSGTYQQSHHSYSSSNH

IFC-2, isoform b 1248 aa

MSLTSTPFSTDDERYSGYTNSAMSPPLQKQLIQEVSLSYSAPYTSTVKPSRHDLPLAFSPYSLDSEIEDLHSHTYSQLSNRSNGNKVE
SAERIYTASRKETEFPAKADPIPYFSAQSAIKAGRESPSYTTRSTPTPRQTPISFDQASTARLDSGLSQNLIVSESTTFEELTTITTS
NNRTTRKKEIKTEMPTVNALTKQFEISSQQEKVVPLKPRKSSNVDYNGNGNSRSQNGTYTPNRSAIVYSPGGILTTTIVDIEPARFGR
RTEEYKIQEEYIVQRYDNGSSPNYQRQEQGYQQQQQHRSREYKTHRQEKQENYNQRSYSATENREVDAPVDTSPIDVVKNVIDRWETK
IGGTTTRFYASDQQLRSVSRVDMNDNVPRSAQEKIIVHQRSESQPTERVIPISPLPHRVEPTRTTQSGGLLQTYEELKTVVVKQSSDQ
TPQKIVPRAQSPVTSPVFKETILSPPPIPTPSPTSDIAEEVLRTDETTQHTAITMHSIVTKEVKQEAKEKINSVTDNAEMKEKVVEIA
KNVEYEWEKKPLTPTPTPIPPQQQEVKQEELKVENKIENDQNEVHLHKFLDRDYRIKTYEEYSTTKRLRDRSQPPERPPTSYSSYSDR
SRSIDRTYERRVLGSGMSRTYSEGRGLASGTYASGFGQLVSGMSSAGAICTTQIRDAREREKREIGLLNDRLADYIEKVRFLEAQNRC
LSHDIDILRNGFSGGGHVSGLFDAEINQAKHILEQTTAHRSTFERDITGLSAEIEVFRKKWLDAVNAVKAHREDHDVDLDRLAKIEAE
ISLFKRKIRIVEEDVIRIRRENDGIYNEIARIKQLTHNEIALKNERSLNVSDLLQRINLLQTENNVRIEQELVFIRRDTTADNRDYFR
HELQAAIRDIRADYEAISIRNRQDIEVWYREQIRKIQDESVRVNPDLYKEELISIRTTVTNVRSRLAEVEGRNFFLERLIDDLKNNEE
AKFFEISLAERDAQIATLRDQCTELSIQMEKLCDNEISLRAEIERYRILLNGANVTTYTSNTHGSGSGIAVGGVVGGSTRVISQTTRT
HSSSNTSYSNVPASRGGYSISGNVGGISVGGTIGSHGASAHATGGIIGSGVQAHRGSVSSLITDKPRDRVHDEKGVDQSGRHFHSWYL
GTISINQVTPSYIELKNICKIRRVDVGGFRIEQSVNGQVLGSAQINVPLILDPQEVVRFNHRHGKYLGQFFMDVDAFDNSTVARTSMY
NYTEPHEERAWFVYLD

IFC-2, isoform c 685 aa

MSLTSTPFSTDDERYSGYTNSAMSPPLQKQLIQEVSLSYSAPYTSTVKPSRHDLPLAFSPYSLDSEIEDLHSHTYSQLSNRSNGNKVE
SAERIYTASRKETEFPAKADPIPYFSAQSAIKAGRESPSYTTRSTPTPRQTPISFDQASTARLDSGLSQNLIVSESTTFEELTTITTS
NNRTTRKKEIKTEMPTVNALTKQFEISSQQEKVVPLKPRKSSNVDYNGNGNSRSQNGTYTPNRSAIVYSPGGILTTTIVDIEPARFGR
RTEEYKIQEEYIVQRYDNGSSPNYQRQEQGYQQQQQHRSREYKTHRQEKQENYNQRSYSATENREVDAPVDTSPIDVVKNVIDRWETK
IGGTTTRFYASDQQLRSVSRVDMNDNVPRSAQEKIIVHQRSESQPTERVIPISPLPHRVEPTRTTQSGGLLQTYEELKTVVVKQSSDQ
TPQKIVPRAQSPVTSPVFKETILSPPPIPTPSPTSDIAEEVLRTDETTQHTAITMHSIVTKEVKQEAKEKINSVTDNAEMKEKVVEIA
KNVEYEWEKKPLTPTPTPIPPQQQEVKQEELKVENKIENDQNEVHLHKFLDRDYRIKTYEEYSTTKRLRDRSQPPERPPTSYSSYSDR
SRSIDRTYERRVLGSGMSRTYSEGNLNFAGLSFKFESSYNMLTVPLSQFLEHQNQTFGTVHFIHYFLCT

IFC-2, isoform d 622 aa

MSTYAAYTTSTYQGRGLASGTYASGFGQLVSGMSSAGAICTTQIRDAREREKREIGLLNDRLADYIEKVRFLEAQNRCLSHDIDILRN
GFSGGGHVSGLFDAEINQAKHILEQTTAHRSTFERDITGLSAEIEVFRKKWLDAVNAVKAHREDHDVDLDRLAKIEAEISLFKRKIRI
VEEDVIRIRRENDGIYNEIARIKQLTHNEIALKNERSLNVSDLLQRINLLQTENNVRIEQELVFIRRDTTADNRDYFRHELQAAIRDI
RADYEAISIRNRQDIEVWYREQIRKIQDESVRVNPDLYKEELISIRTTVTNVRSRLAEVEGRNFFLERLIDDLKNNEEAKFFEISLAE
RDAQIATLRDQCTELSIQMEKLCDNEISLRAEIERYRILLNGANVTTYTSNTHGSGSGIAVGGVVGGSTRVISQTTRTHSSSNTSYSN
VPASRGGYSISGNVGGISVGGTIGSHGASAHATGGIIGSGVQAHRGSVSSLITDKPRDRVHDEKGVDQSGRHFHSWYLGTISINQVTP
SYIELKNICKIRRVDVGGFRIEQSVNGQVLGSAQINVPLILDPQEVVRFNHRHGKYLGQFFMDVDAFDNSTVARTSMYNYTEPHEERA
WFVYLD

IFC-2, isoform e 502 aa

MSTYAAYTTSTYQGRGLASGTYASGFGQLVSGMSSAGAICTTQIRDAREREKREIGLLNDRLADYIEKVRFLEAQNRCLSHDIDILRN
GFSGGGHVSGLFDAEINQAKHILEQTTAHRSTFERDITGLSAEIEVFRKKWLDAVNAVKAHREDHDVDLDRLAKIEAEISLFKRKIRI
VEEDVIRIRRENDGIYNEIARIKQLTHNEIALKNERSLNVSDLLQRINLLQTENNVRIEQELVFIRRDTTADNRDYFRHELQAAIRDI
RADYEAISIRNRQDIEVWYREQIRKIQDESVRVNPDLYKEELISIRTTVTNVRSRLAEVEGRNFFLERLIDDLKNNEEAKFFEISLAE
RDAQIATLRDQCTELSIQMEKLCDNEISLRAEIERYRILLNGANVTTYTSNTHGSGSGIAVGGVVGGSTRVISQTTRTHSSSNTSYSN
VPASRGGYSISGNVGGISVGGTIGSHGASAHATGGIIGSGVQAHRGSGTYQQSHHSYSSSNH

Supplementary Figure S1. Graphic representation of gene models proposed for the *ifc-2* gene locus and their corresponding protein sequences. (a) The *ifc-2* gene consists to two partially overlapping transcriptional units. Promoter 1 drives transcription starting at exon 1 and generates variants *ifc-2a*, *ifc-2b* and *ifc-2c*, which encode polypeptides with different carboxytermini. Promoter 2 drives transcription starting at exon 11 and generates variants *ifc-2d* and *ifc-2e*, which encode polypeptides with different carboxytermini in the same way as *ifc-2a* and *ifc-2b*, respectively. Differentially translated regions are highlighted in red. The position and types of tags integrated into the *ifc-2* locus are shown for alleles *qp110* and *kc16*. (b) shows the amino acid sequences for all IFC-2 isoforms. Identical sequences are color-coded for better comparison.

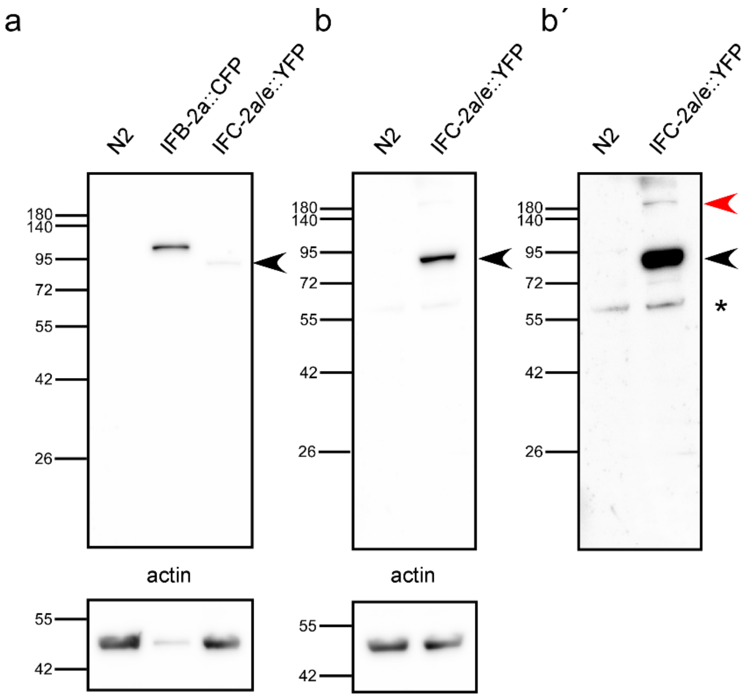

Supplementary Figure S2. Immunoblot detects polypeptides IFC-2a::YFP and IFC-2e::YFP produced from *ifc-2* allele *kc16*. Cytoskeletal protein extracts were prepared from wild-type N2, *ifb-2a::cfp* reporter strain BJ49 and strain BJ316 containing a YFP-encoding cassette that is inserted into the 3'-end of *ifc-2a* and *ifc-2e*. Polypeptides were separated by 10% SDS gel electrophoresis, transferred onto PVDF membrane and reacted with anti-GFP antibodies. Position and size in kDa of co-electrophoresed molecular weight markers are shown at left of each blot. One can detect a major species expected for the IFC-2e::YFP fusion (calculated size 83 kDa; black arrowhead in a, b, b') and a minor species expected for the IFC-2a::YFP fusion (calculated size 155 kDa), which is only visible after long exposure (red arrowhead in b'). Since IFB-2a::CFP is produced at much higher levels, 15 times less BJ49 lysate was loaded. Note that the apparent molecular weights are consistently higher than the calculated molecular weights of all IF polypeptides (calculated molecular weight of IFB-2a::CFP 89 kDa). The asterisk marks a non-specific signal that is present both in wild type and in BJ316.

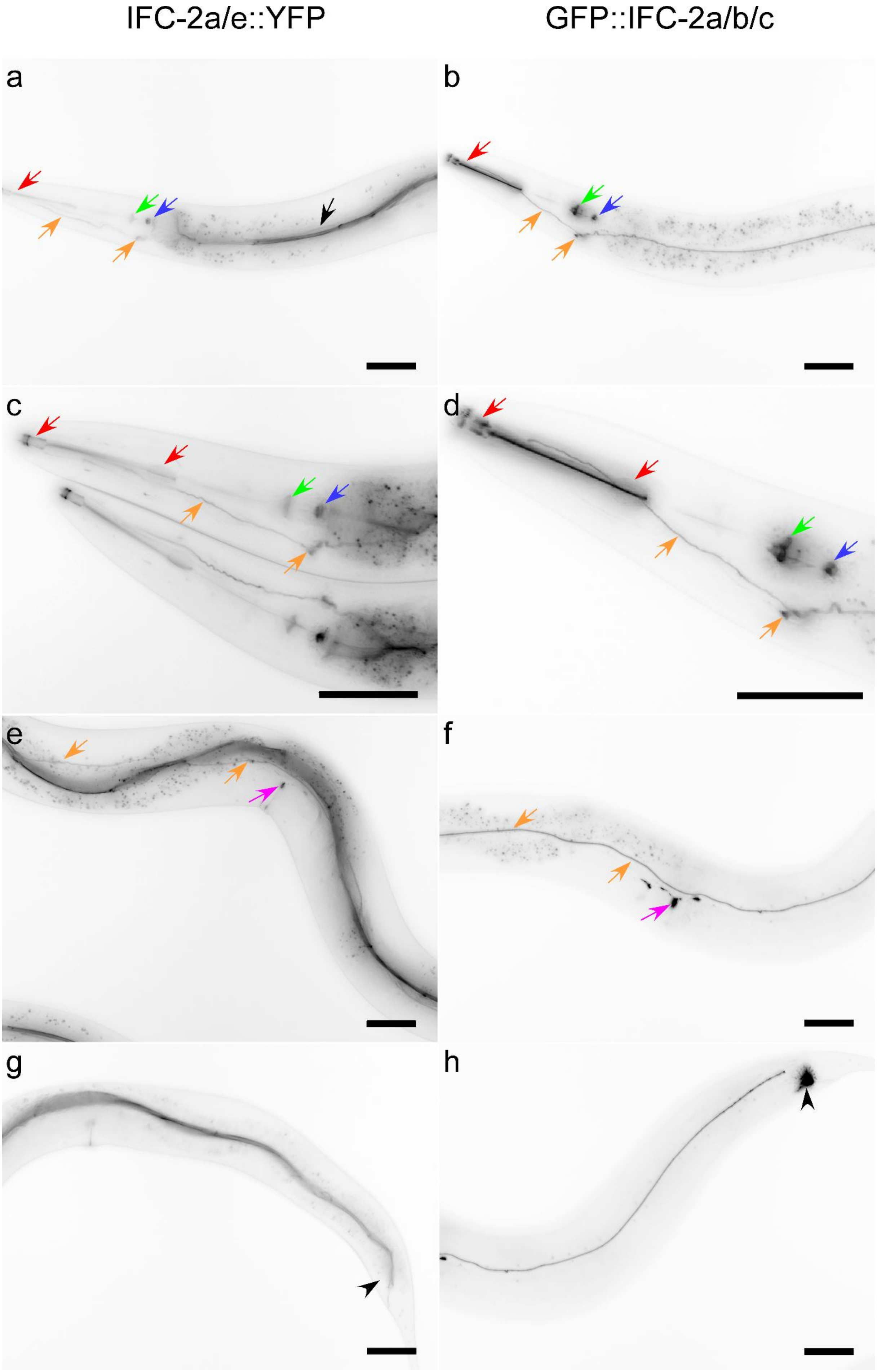

IFC-2a/e::YFP
GFP::IFC-2a/b/c
a
b
c
d
e
f
g
h

Supplementary Figure S3. Only promoter 2 of the *ifc-2* gene locus is active in the intestine. The maximum intensity fluorescence images show a comparison of the fluorescence in strain BJ316 producing tagged IFC-2a/e::yfp variants from allele *ifc-2(kc16)* (left column) and BK531 producing an aminoterminally 3xFLAG- and GFP-tagged IFC-2a/b/c reporter (corresponding to GFP::EXC-2 in [1]; right column). While IFC-2a/e::YFP is localized in the intestine (black arrow), the excretory canal (orange arrow), corpus and posterior bulb of the pharynx (red and green arrow, respectively), pharyngeal-intestinal valve (blue arrow) and interfacial uterine cells (pink arrow), no intestinal expression can be observed for IFC-2a/b/c, which instead presents a very prominent localization in the intestinal-rectal valve (similar regions marked with black arrowheads). Scale bars: 50 µm.

a *ifb-2(kc14)*

cgacttcatcATG|TCG|GCG|GTT|AGT|TAT|TCG|ATG|CAC|AGA|ACC|ACC|ACA|A[T]C|TAC|ATC|CTC|CTC|ATC|ACA|CGG|AGG|TGT|CTC|AGC|CGG|CCA|TGC|CGC|**TGA**|GGAGTTC
GTTGCCTCTGCCGAGCGCGAGAAGCAAGAGATGCAGCAGTTGAACTCTCGCCTTGAAGTTTACATCAGCCGTGTCCGTCAACTTGAGGATCGTAACAAGGAGCTTGTGATTGAATTGGACACCCTCCGAGGATCACTTG
GAAATGACATCGGACAGATCAAGTTCAAATTCAACGATTCGTTGGTCAAAGTTCGCCGTGAGATCTCAGAAGCTCATTCTGGAACTATTGGAGTTGAAGTTAAGGTCGACAGATTGAGAGATGATTTGAATGACTACAG
ACACAGATATGAAGAGGCCCGTCGTGAAGTTGAGCGTGAGAAGACCACTTGGGGAGGAGCTATTTCACAAGCACAAGCTGAGCTTGACACAAACAAGTCCCGCTATGCCGCCATTTTGGACGAGGAAAAGAGACTTTAT
GCTGAACAAGATCAACTCTATCTTCAATTGGCTGCCGCCAAGGATGAGCTTGACGCCGCAATTGTTGATCGCCGCCGCCTTCAAGCTGAGGAAGATGACCTCAAGATTGAGCTGGAATTCTTGGGAAGAATCCACTCTC
AAGAAATCACTGAGCTTCGCACTCTTCTTGCTCAAGCTCCAGCCGACACAAGAGAGTTCTTCAAGAATGAATTGGCTCTTGCTATCAGAGAAATCAAGGCTGAATACGACAAGATCATCCAAACCACCAGAGTTGATCT
TGAGACAATCTTCCAGAGCAAGATTAGCGCCGTCGAATCATCAATCGTCTCCAAGAACGAGGCTGCCGTTTTCCGTCAAGAGGAGATCAGAAAGATGAACGAGAGCATCACCACATTGCGAGCCAAGCTCAGCGAGCTC
GAAGCCCGTAACTCTGCTTTGGAGAGAGAAGCCAACACACTCCAGATTCAACTCGGAGAAGATCAAAGAGCTTACGAATCAGAGCTTCACAAGAGAGACAATGCCCTCCGATTTATGCGTGAAGACTGCCAGACTTTGA
TTGCTGAACTTCAAGCTCTTCTTAACACCAAACAAACTTTGGATACTGAGATTGCCATCTACAGAAAGCTTGTCGAGTCCGAGGAAGGAAGATTCACTCATGTTGGACAGGGGGTCGTTGTCGCCCAGCAGGAGACTAC
AAGATTGGTACCAGTCGAGCAAGATCACTGGGACTCTGGAGAGGTTCAAACTCGTTCCTCATTCAAGAGACACGCTAAGGGAAATGTGAGCATCGTTGAGTGCGATCCACAAGGAAAGTACATCATTCTTGAAAACACC
AGTGGATCAGTTGCCGAAGATGTAAGCAACTTTGAAATCCGTCGTGTTATTGATGGAGTTCAAGCCTTCGTTTTCCGTCTCCCATCTCACTTAGTTATTCAACAACACGGACATCTTAAGATCTACGGACGCAATTCAG
GAGGAATTAACTCACCACCAGACTCAATTGTGATGGAATCTCACCCATCATGGGGACAAGGAGGACAAGTTGAAACATTCTTGTACAATAGTCACGGAATTGAGAAGGCTTCGCACATCCAGACGACGGTCGCTTCTTC
CCGTTAAgaaatctccatcatctaatcgatttctttttcgatgaatttaaatttattctgaaatgcccattgaatcctttccttgctgtcttcactcccaaaatcattcgacgtacatacttgctacattccaatccaa
ttgtttttgaaataaattgttcaatattg

b *ifc-2(kc15)*

gagaaacggagaaacacagcgaccagcgccaaactgattacagttggcattatgttgaaggcataggtggagtcgcaagggttgcctataaaacattgtctaatcagtgccattttctcatttttttatcattattaact
tctaaaatcgacaaaaaaaaaccaaaaaATG|TCG|ACT|TAC|GCG|GCC|TAT|ACT|ACT|TCT|ACT|TAT|CAA|GGT|CGT|GGA|CTT|GCC|AGT|GGA|ACA|TAT|GCT|~~TCTGGCTTTGGCCAGCTTG~~
~~TCAGTGGAATGTC~~ATC|TGC|TGG|AGC|CAT|CTG|CAC|AAC|CCA|AAT|CCG|**TGA**|CGCGCGTGAACGTGAGAAGAGAGAAATTGGTCTCTTGAACGATCGTCTCGCGGATTACATTGAGAAGGTTCGTTTTCT
GGAAGCTCAAAACCGATGCCTCAGCCACGATATTGATATTCTTCGCAACGGATTCAGCGGTGGAGGTCATGTATCTGGACTCTTTGACGCTGAGATCAACCAAGCAAAGCATATTCTTGAGCAAACAACCGCTCACCGC
TCCACATTTGAAAGAGACATCACTGGTCTCTCCGCGGAGATTGAAGTGTTCAGAAAGAAATGGCTGGACGCTGTCAACGCCGTCAAAGCTCATCGTGAGGACCATGATGTGGATTTGGACAGACTTGCCAAGATTGAAG
CTGAAATTTCACTTTTCAAAAGAAAGATCCGTATTGTTGAGGAAGACGTCATCAGAATTCGCAGAGAAAATGACGGAATTTACAATGAAATCGCCAGAATCAAGCAGCTTACCCATAACGAAATTGCTCTGAAGAATGA
GCGCAGCTTGAATGTCTCCGATCTTCTTCAGCGCATCAACCTCCTACAAACGGAGAACAACGTCAGAATCGAGCAAGAACTTGTCTTCATCCGCCGTGACACCACTGCCGACAACCGTGATTACTTCAGACATGAGCTT
CAAGCCGCCATTCGTGACATCCGTGCCGATTATGAAGCCATCAGCATCCGCAACCGTCAAGACATTGAAGTGTGGTACCGTGAGCAAATCAGAAAGATTCAGGACGAATCAGTTCGCGTCAACCCAGACCTCTACAAGG
AAGAACTCATCTCCATCCGCACGACCGTGACAAATGTCCGAAGCAGACTTGCCGAGGTTGAAGGAAGAAACTTCTTCCTTGAGCGGCTCATTGATGACCTAAAGAACAACGAGGAAGCCAAGTTCTTCGAGATCAGCCT
TGCCGAGAGAGACGCCCAGATTGCGACCCTCCGTGACCAGTGCACAGAGCTCAGCATTCAAATGGAGAAACTCTGTGACAACGAAATTTCCCTCCGCGCCGAAATCGAGCGCTATCGCATCCTCCTCAACGGAGCAAAC
GTGACAACGTACACCTCCAATACGCACGGAAGCGGCTCTGGAATCGCAGTCGGTGGTGTTGTTGGAGGATCAACCCGCGTTATATCTCAAACCACGAGAACCCACAGCAGCAGCAATACGAGCTACTCAAATGTCCCTG
CCTCTAGAGGCGGCTACTCCATCAGTGGGAACGTTGGAGGAATTTCAGTTGGAGGAACCATTGGATCGCACGGAGCTTCGGCCCATGCGACAGGAGGAATCATTGGCTCTGGAGTTCAGGCGCACAGAGGAAGTGGTAC
GTATCAACAGAGCCA**T**CACTCCTACTCCTCTTCAAATCACCGGTCCATGGTGAGCAAGGGCGAGGAGCTGTTCACCGGGGTGGTGCCCATCCTGGTCGAGCTGGACGGCGACGTAAACGGCCACAAGTTCAGCGTGTCC
GGCGAGGGCGAGGGCGATGCCACCTACGGCAAGCTGACCCTGAAGTTCATCTGCACCACCGGCAAGCTGCCCGTGCCCTGGCCCACCCTCGTGACCACCTTCGGCTACGGCCTGCAGTGCTTCGCCCGCTACCCCGACC
ACATGAAGCAGCACGACTTCTTCAAGTCCGCCATGCCCGAAGGCTACGTCCAGGAGCGCACCATCTTCTTCAAGGACGACGGCAACTACAAGACCCGCGCCGAGGTGAAGTTCGAGGGCGACACCCTGGTGAACCGCAT
CGAGCTGAAGGGCATCGACTTCAAGGAGGACGGCAACATCCTGGGGCACAAGCTGGAGTACAACTACAACAGCCACAACGTCTATATCATGGCCGACAAGCAGAAGAACGGCATCAAGGTGAACTTCAAGATCCGCCAC
AACATCGAGGACGGCAGCGTGCAGCTCGCCGACCACTACCAGCAGAACACCCCCATCGGCGACGGCCCCGTGCTGCTGCCCGACAACCACTACCTGAGCTACCAGTCCGCCCTGAGCAAAGACCCCAACGAGAAGCGCG
ATCACATGGTCCTGCTGGAGTTCGTGACCGCCGCCGGGATCACTCTCGGCATGGACGAGCTGTACAAG**TAA**

c *ifp-1(kc18)*

cgcATG|G~~ATTCCGCTAACGCTAGGGATTGTCTA~~CT|ACA|TCT|GGC|AAG|AGC|**TAA**|GCTCTCTGAGCGACAAGACCTTGTTCAGCTCAACGATCAGTTTGTGGATATCATCGAACATGTCCACTATATGGAAGC
TGAGCACACTGCTTTGGAACACGACTACAATTTGCTAAAAAGTGGAGTCCAGAGCGACAGTAGTGGAATTAATGAAATTTACAATGTTGAGATCAGGACTGTCAGATCGGGAATTGAAGAAATTAACCGAAGCCGGCAC
GAACTACTAAATGAGCAAACCCAGTTGTCCCATCAAGTCAAAGAAGCCGAACAACTTTGGAGACACACTGCAAAATCAGCTTTGGGGGTTCCCAAAGAGGTTGATGATGAGTTCCATAGAATCTGTCATATCAAGATGG
AAGATTGTATCACTAAGAGAAGAATCAAGTATATGGAGGATAAGCTCAGACTGATCAAACAGAACAATGGAAGAATTTTTGAGCATATTAATTTGATGAGAATGAGAAAAGATCAAGCTGTCAGTCTGCAACAAGAATA
TCTTCTTCGCAAGAATGAGTTGTTGCACAGTATCCGAAATATGGAGGAAGACAACAAAAAGATTATCATGAATGAGCACAAATACTTTGTTCGTGATCGCAATGCGGATCGTCACGTCTTCCGCGATCAACTTCGCAAG
GCAATTGCTGATATTCGTGCTGATTATGAGGCTAACAGATTGAGAAACGAAGAAGAAATTCGAATTCGCTTGGAAAGGGAAATCCATCGAATGAATACCACTATGCCAGGAGTAGTTTGCTATGACAAACTGAGAGAGG
AGCTTTCGATTGTTAAGAACAATCTTTCTGGATTGCAGAAACAAGTTTCTGAAGTTGAAATCAGAAACAATTCTCTCACTCAACAAATTGAATTCTATCGTCTGGAAATTTCGGAAAATAACAAGCTTTTCGAGGATAC
TTTGGAATTCAAAATAAAGGAAATTGAGAAGATGAGAGAGCAATGCACTGCCATCAGTGTTGAATTGGAGAAACTTTGTGACTTAAACATTGACTTGCAAAAGGAAATCGCAAAATATCGGGAACTTCTCGACAGATCT
GGAGATCCTCGTGCAAATGCGCCAAGTACTCAAGTTATAGCAAGCGCGCTTAGTCGACAGAACTACAGCAGCATTTCTGACCACCATAGCCGAACTTCTTCTGGAATAGTTCAGCACACTGCTCATCACGCTGTGAGAG
ATTCCTCGTCGTCCAGAACTGGTACCACTATTCATGAGACCATCACTCACACGCCAATTCCAATTCCTGCTACACTTCCTGTTCAACCAATTCGTGAATATACTTATTCGAGGGACGCGAGCCCGATTCGTCCATCATA
TACTCCGTACCAGCAGGAGTCTCGTGCTGATAGCCGATCTTCTCTCAGGGAGAGAATTCAAATCGAGACTCTCCAAGGCACACTTCCGGTTCTGCCAAGCCATTCGGTTGAAACAATTCGTGCTTCAGATAACACCCGA
TTAGTGAGAGACCTTAGCGCGGGAGGTCAGATTAACACAACACAGATTAACAATCCATACGCGTCTCGTACTCCAACTTCATCGGTCAATGATAGGATTGCAAGCGAACGTCGTGATAGTGAAATTCGTCGCAACGATG
CTTTACATCAACCTTATCCAATTGGTGGTGCCGTTCCTCGAGTTGAGCCTACAATTTTTAATGAACGTGCTCCCGGTTACACTCATATTAATAGCAACTTCCCGCTAATTACGCCAGTGACTGAAGGAGCTACTGTCCC
CTCATACTACGATACTTTTAATCGGAACTCGAGCCAAAGAGGGCCACATCATTCGAGCTATCATGCAGCAACAGGCTCAGTGAGCAATAGCATCTCCACTACTCTACTCAATGAAGGATCAATTGAACATAGCAGTAGT
TCCGATAGTGTTGACGAGGACAACTTCCAGAGATTCACTAGATGGTACAAGGGGAGAGTGAAGATTTCGGATGTGACTGCAGATTTTGTTCAACTTGTAAATAGAAGCTCGAAAAAATCGGCAGACGTTGGTGGCTTCA
AACTTATTCACGAGTTTGGAAACAAGAGCGTATATGTTGATCTTCCAGTTGGATTGATCCTTGCACCCAAGGATAGCTTGAAGATCTACGCTCGTGGAGCAACTCATGAAAGAGGAGCAGTTATTGCTGAAATTGACTT
CTTCGATACCACCATCCATACCAATACATCAATCAGAAACACGAATAATGAGGTGAAATCGTGGTTTGTTTACACGAGCAACACTGAAATCGGAGATGCAGACCATCATCATCATTGAtacattactaatgccaaattt
ctgataacatgataattctaacaacaaatatatattttattttctcttgttttcatttttcacactttttatcaagaaacttctactcgttgcccctatttttttccactcacttcttgaaaaaggaaataattcttta
attcaatcccagtaattcgtatttctttcatattctgaatttaaaataaacctattact

d *kcIs41[ifp-1p::ifp-1[16-19del]::egfp *kcIs40]*

cgcATG|GAT|TCC|GCT|AAC|~~GCTA~~GGG|ATT|GTC|TAC|TAC|ATC|TGG|CAA|GAG|CTA|AGC|TCT|CTG|AGC|GAC|AAG|ACC|TTG|TTC|AGC|TCA|ACG|ATC|AGT|TTG|TGG|ATA|TCA|
TCG|AAC|ATG|TCC|ACT|ATA|TGG|AAG|CTG|AGC|ACA|CTG|CTT|TGG|AAC|ACG|ACT|ACA|ATT|TGC|**TAA**|AAAGTGGAGTCCAGAGCGACAGTAGTGGAATTAATGAAATTTACAATGTTGAGAT
CAGGACTGTCAGATCGGGAATTGAAGAAATTAACCGAAGCCGGCACGAACTACTAAATGAGCAAACCCAGTTGTCCCATCAAGTCAAAGAAGCCGAACAACTTTGGAGACACACTGCAAAATCAGCTTTGGGGGTTCCC
AAAGAGGTTGATGATGAGTTCCATAGAATCTGTCATATCAAGATGGAAGATTGTATCACTAAGAGAAGAATCAAGTATATGGAGGATAAGCTCAGACTGATCAAACAGAACAATGGAAGAATTTTTGAGCATATTAATT
TGATGAGAATGAGAAAAGATCAAGCTGTCAGTCTGCAACAAGAATATCTTCTTCGCAAGAATGAGTTGTTGCACAGTATCCGAAATATGGAGGAAGACAACAAAAAGATTATCATGAATGAGCACAAATACTTTGTTCG
TGATCGCAATGCGGATCGTCACGTCTTCCGCGATCAACTTCGCAAGGCAATTGCTGATATTCGTGCTGATTATGAGGCTAACAGATTGAGAAACGAAGAAGAAATTCGAATTCGCTTGGAAAGGGAAATCCATCGAATG
AATACCACTATGCCAGGAGTAGTTTGCTATGACAAACTGAGAGAGGAGCTTTCGATTGTTAAGAACAATCTTTCTGGATTGCAGAAACAAGTTTCTGAAGTTGAAATCAGAAACAATTCTCTCACTCAACAAATTGAAT
TCTATCGTCTGGAAATTTCGGAAAATAACAAGCTTTTCGAGGATACTTTGGAATTCAAAATAAAGGAAATTGAGAAGATGAGAGAGCAATGCACTGCCATCAGTGTTGAATTGGAGAAACTTTGTGACTTAAACATTGA
CTTGCAAAAGGAAATCGCAAAATATCGGGAACTTCTCGACAGATCTGGAGATCCTCGTGCAAATGCGCCAAGTACTCAAGTTATAGCAAGCGCGCTTAGTCGACAGAACTACAGCAGCATTTCTGACCACCATAGCCGA
ACTTCTTCTGGAATAGTTCAGCACACTGCTCATCACGCTGTGAGAGATTCCTCGTCGTCCAGAACTGGTACCACTATTCATGAGACCATCACTCACACGCCAATTCCAATTCCTGCTACACTTCCTGTTCAACCAATTC
GTGAATATACTTATTCGAGGGACGCGAGCCCGATTCGTCCATCATATACTCCGTACCAGCAGGAGTCTCGTGCTGATAGCCGATCTTCTCTCAGGGAGAGAATTCAAATCGAGACTCTCCAAGGCACACTTCCGGTTCT
GCCAAGCCATTCGGTTGAAACAATTCGTGCTTCAGATAACACCCGATTAGTGAGAGACCTTAGCGCGGGAGGTCAGATTAACACAACACAGATTAACAATCCATACGCGTCTCGTACTCCAACTTCATCGGTCAATGAT
AGGATTGCAAGCGAACGTCGTGATAGTGAAATTCGTCGCAACGATGCTTTACATCAACCTTATCCAATTGGTGGTGCCGTTCCTCGAGTTGAGCCTACAATTTTTAATGAACGTGCTCCCGGTTACACTCATATTAATA
GCAACTTCCCGCTAATTACGCCAGTGACTGAAGGAGCTACTGTCCCCTCATACTACGATACTTTTAATCGGAACTCGAGCCAAAGAGGGCCACATCATTCGAGCTATCATGCAGCAACAGGCTCAGTGAGCAATAGCAT
CTCCACTACTCTACTCAATGAAGGATCAATTGAACATAGCAGTAGTTCCGATAGTGTTGACGAGGACAACTTCCAGAGATTCACTAGATGGTACAAGGGGAGAGTGAAGATTTCGGATGTGACTGCAGATTTTGTTCAA
CTTGTAAATAGAAGCTCGAAAAAATCGGCAGACGTTGGTGGCTTCAAACTTATTCACGAGTTTGGAAACAAGAGCGTATATGTTGATCTTCCAGTTGGATTGATCCTTGCACCCAAGGATAGCTTGAAGATCTACGCTC
GTGGAGCAACTCATGAAAGAGGAGCAGTTATTGCTGAAATTGACTTCTTCGATACCACCATCCATACCAATACATCAATCAGAAACACGAATAATGAGGTGAAATCGTGGTTTGTTTACACGAGCAACACTGAAATCGG
AGATGCAGACCATCATCATGAAGTGCATACCAATCAGGACCCGCTGGATGAAGTCCACACAAACCAAGATCCACTCGATACTAGTATGAGTAAAGGAGAAGAACTTTTCACTGGAGTTGTCCCAATTCTTGTTGAATTA
GATGGTGATGTTAATGGGCACAAATTTTCTGTCAGTGGAGAGGGTGAGGGTGATGCAACATACGGAAAACTTACCCTTAAATTTATTTGCACTACTGGAAAACTACCTGTTCCATGGGTAAGTTTAAACATATATATAC
TAACTAACCCTGATTATTTAAATTTTCAGCCAACACTTGTCACTACTTTCTGTTATGGTGTTCAATGCTTCTCGAGATACCCAGATCATATGAAACAGCATGACTTTTTCAAGAGTGCCATGCCCGAAGGTTATGTACA
GGAAAGAACTATATTTTTCAAAGATGACGGGAACTACAAGACACGTAAGTTTAAACAGTTCGGTACTAACTAACCATACATATTTAAATTTTCAGGTGCTGAAGTCAAGTTTGAAGGTGATACCCTTGTTAATAGAATC
GAGTTAAAAGGTATTGATTTTAAAGAAGATGGAAACATTCTTGGACACAAATTGGAATACAACTATAACTCACACAATGTATACATCATGGCGGACAAACAAAAGAATGGAATCAAAGTTGTAAGTTTAAACATGATTT
TACTAACTAACTAATCTGATTTAAATTTTCAGAACTTCAAAATTAGACACAACATTGAAGATGGAAGCGTTCAACTAGCAGACCATTATCAACAAAATACTCCAATTGGCGATGGCCCTGTCCTTTTACCAGACAACCA
TTACCTGTCCACACAATCTGCCCTTTCGAAAGATCCCAACGAAAAGAGAGACCACATGGTCCTTCTTGAGTTTGTAACAGCTGCTGGGATTACACATGGCATGGATGAACTATACAAATCCGGAAGTTCCTATTCTCTA
GAAAGTATAGGAACTTCCCTCGAGGATTATAAAGACCACGATGGAGACTATAAAGATCATGACATTGACTACAAGGATGACGACGACAAGTGAtacattactaatgccaaattt

Supplementary Figure S4. Genetic alterations of alleles *ifb-2(kc14), ifc-2(kc15), ifp-1(kc18) and kcIs41[ifp-1p::ifp-1[16-19del]::egfp *kcIs40]*. (a) shows the spliced gene model of *ifb-2* isoform a, (b) the gene model of *ifc-2e* with carboxyterminal *yfp* tag highlighted in green (*ifc-2(kc16)*; linker in blue, silent mutation of the PAM motif in purple, bold and underlined), (c) the spliced gene model of *ifp-1* isoform a and (d) the gene model of integrated *ifp-1p::ifp-1::egfp* reporter fusion (*kcIs40*). Note the carboxyterminal *egfp* tag with artificial introns highlighted in green. Exons shown in alternating colors, non-translated regions in grey letters and translational start codon in green according to www.wormbase.org (Release WS272). (a) Mutant allele *ifb-2(kc14)* contains a thymidine insertion at position 41 (bracketed in red) leading to a premature stop in the second exon (TGA underlined) resulting in a truncated 29 amino acid-long protein encompassing only 13 of the most aminoterminal amino acids of IFB-2a. The mutation also affects all other known IFB-2 isoforms, which share the same start codon and the aminoterminal part encoded by the first five exons. (b) Mutant allele *ifc-2(kc15)* contains a 32 base pair deletion in the first exon (crossed out) causing a premature stop (TGA underlined) resulting in a truncated 34 amino acid-long protein encompassing only 23 of the most aminoterminal amino acids of IFC-2d and e. (c) Deletion allele *ifp-1(kc18)* carries a 26 base pair deletion in the first exon (crossed out) causing a premature stop (TAA underlined) leading to a truncated 6 amino acid-long protein encompassing no amino acids of all IFP-1 isoforms. (d) *kcIs41[ifp-1p::ifp-1[16-19del]::egfp *kcIs40]* contains a four base pair deletion after position four causing a frameshift in the second exon leading to a truncated 52 amino acid protein encompassing only four of the most aminoterminal amino acids of IFP-1::EGFP fusion protein.

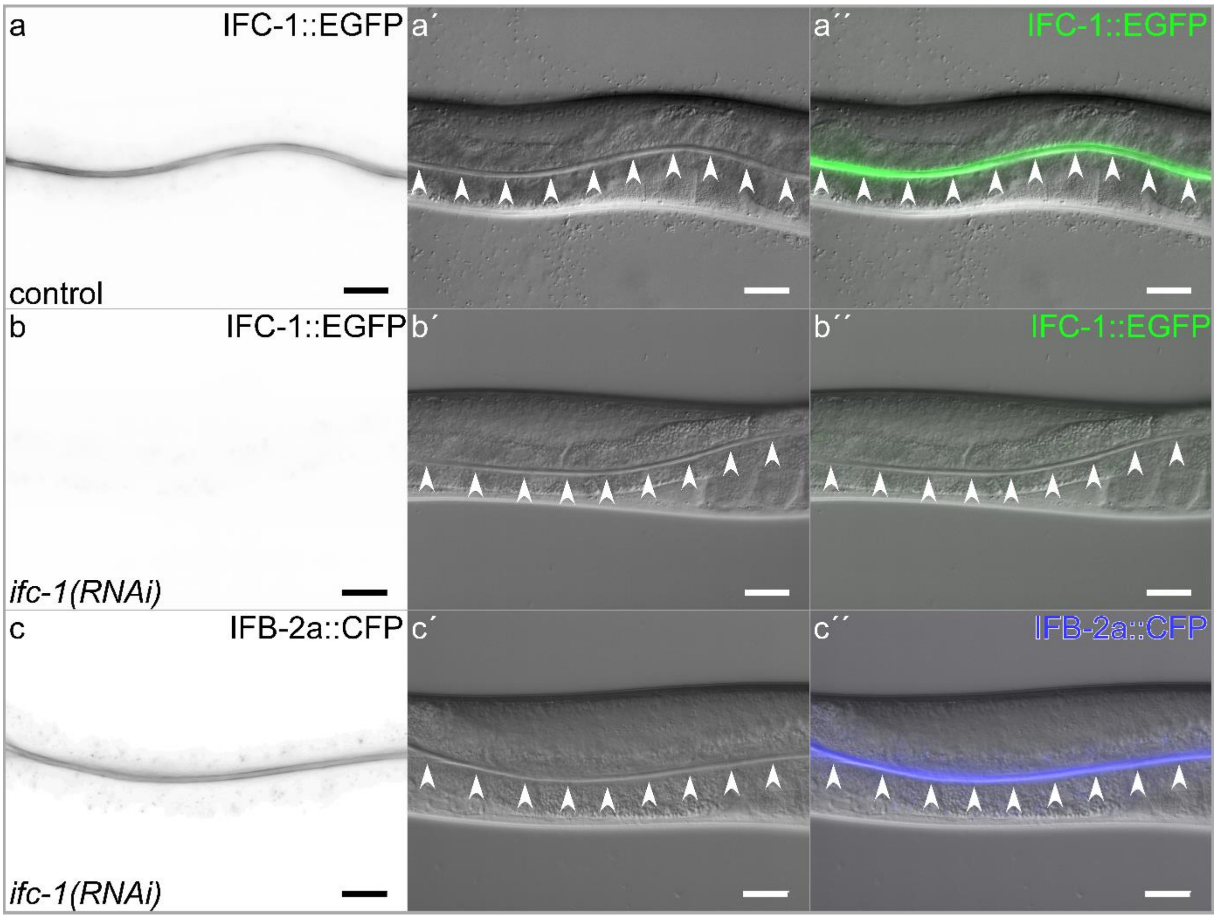

Supplementary Figure S5. IFC-1 is not essential for intestinal lumen maintenance and IFB-2 distribution. (a-c'') The micrographs show a comparison of the intestinal IFC-1::EGFP fluorescence encoded by extrachromosomal reporter *ifc-1::egfp* in control strain BJ324 (a) and *ifc-1(RNAi)* animals (b; corresponding interference contrast images in a', b'; merged images in a'', b'') and the IFB-2a::CFP fluorescence in *ifc-1(RNAi)* animals of strain BJ49 (c; corresponding interference contrast image in c'; merged image in c''). Note that *ifc-1(RNAi)* efficiently downregulates IFC-1::EGFP but does not affect lumen morphology and IFB-2a::CFP distribution. Scale bars: 25 µm.

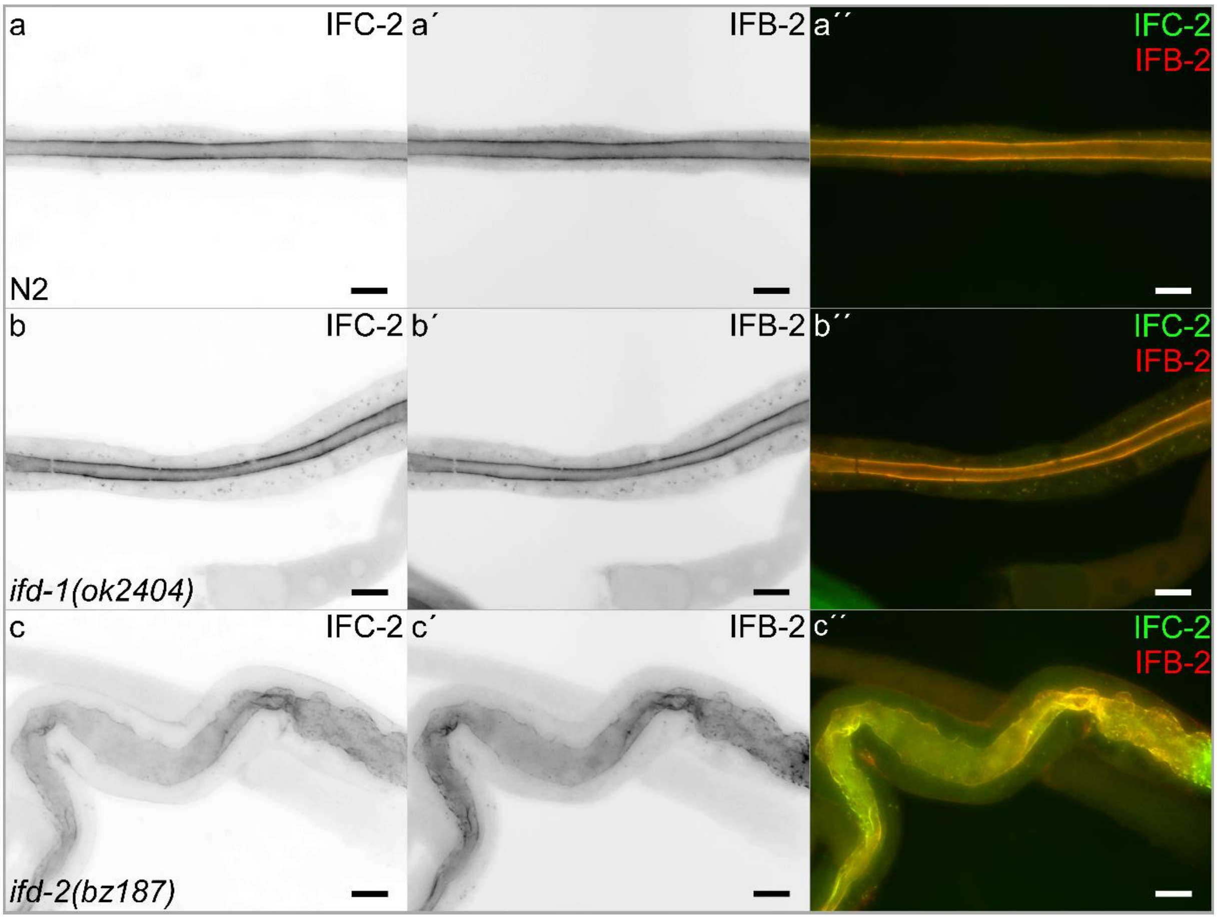

Supplementary Figure S6. Loss of function mutation of *ifd-1* does not affect intestinal lumen morphogenesis whereas loss of function mutation of *ifd-2* induces luminal widening and cytoplasmic invaginations. The micrographs depict a comparison of anti-IFC-2 (a, b, c) and anti-IFB-2 immunostaining (a', b', c'; merged images in a'', b'', c'') in fixed intestines isolated from adult wild-type N2 (a-a'') and mutant strains BJ374 and BJ375 carrying mutant allele *ifd-1(ok2404)* (b-b'') and *ifd-2(bz187)* (c-c''), respectively. Scale bars: 20 µm.

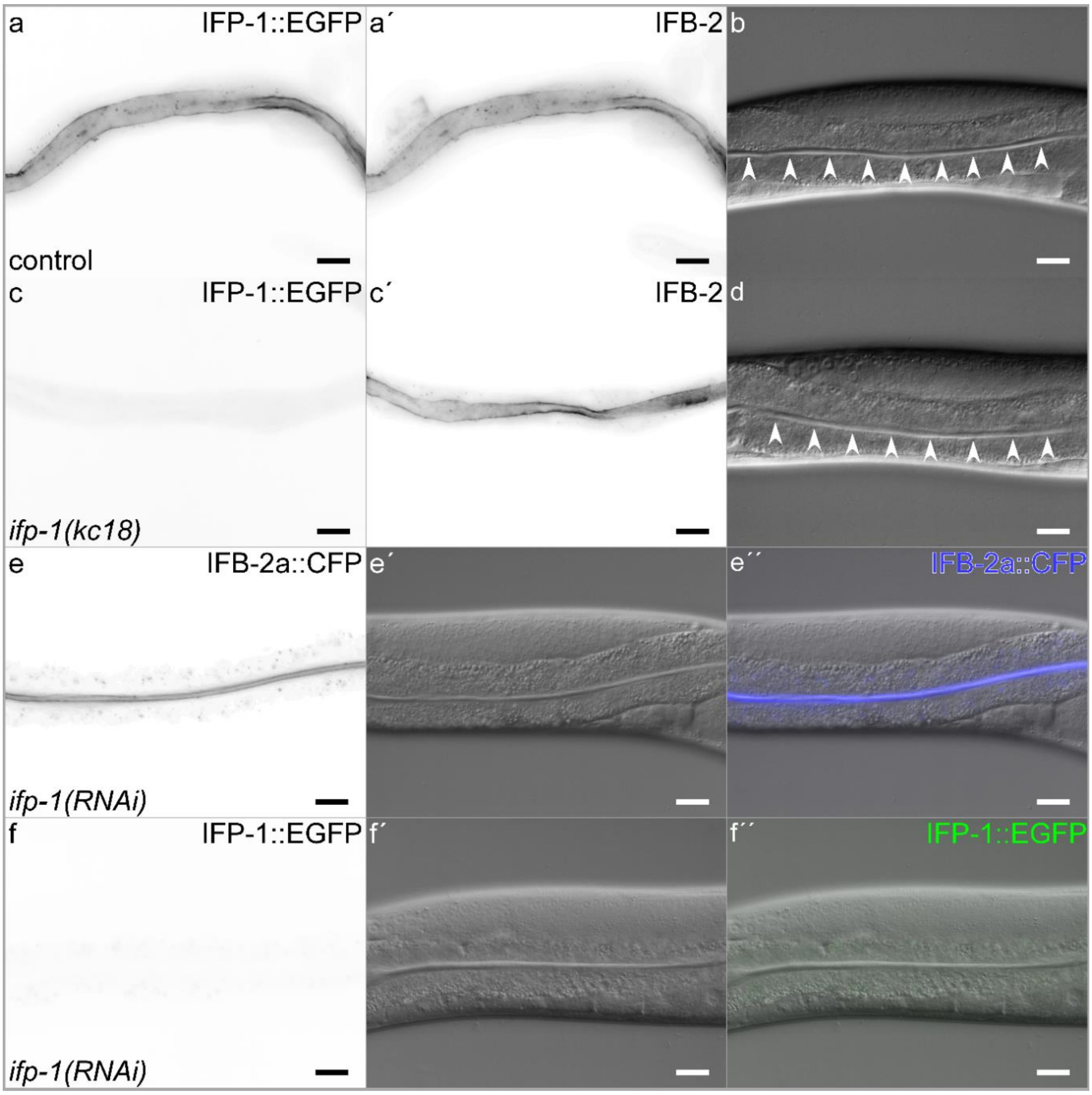

Supplementary Figure S7. IFP-1 is not essential for intestinal lumen maintenance and IFB-2 distribution. (a-d) The micrographs show a comparison of the intestinal IFP-1::EGFP fluorescence (a, c) that is produced from the integrated fosmid-derived reporter *ifp-1::egfp* and anti-IFB-2 immunostaining (a', b') in isolated intestines either in a wild-type control background (strain BJ312) or mutant *ifp-1(kc18)* background (strain BJ320) together with a comparison of the intestinal lumen morphology detected by interference contrast microscopy (b, d). The CRISPR/cas9-mediated *ifp-1* mutations efficiently abrogates all IFP-1::EGFP fluorescence (compare a and c) but does not affect IFB-2 immunofluorescence (compare a', c'). Note also that interference contrast images do not reveal morphological changes in the mutant intestines (b, d). (e-f'') The micrographs depict IFB-2a::CFP and IFP-1::EGFP fluorescence together with corresponding differential interference contrast images (e', f'; merged images in e'', f'') in reporter strains BJ49 (e-e'') and BJ312 (f-f'') after treatment with *ifp-1(RNAi)*. Scale bars: 20 µm in a-f''.

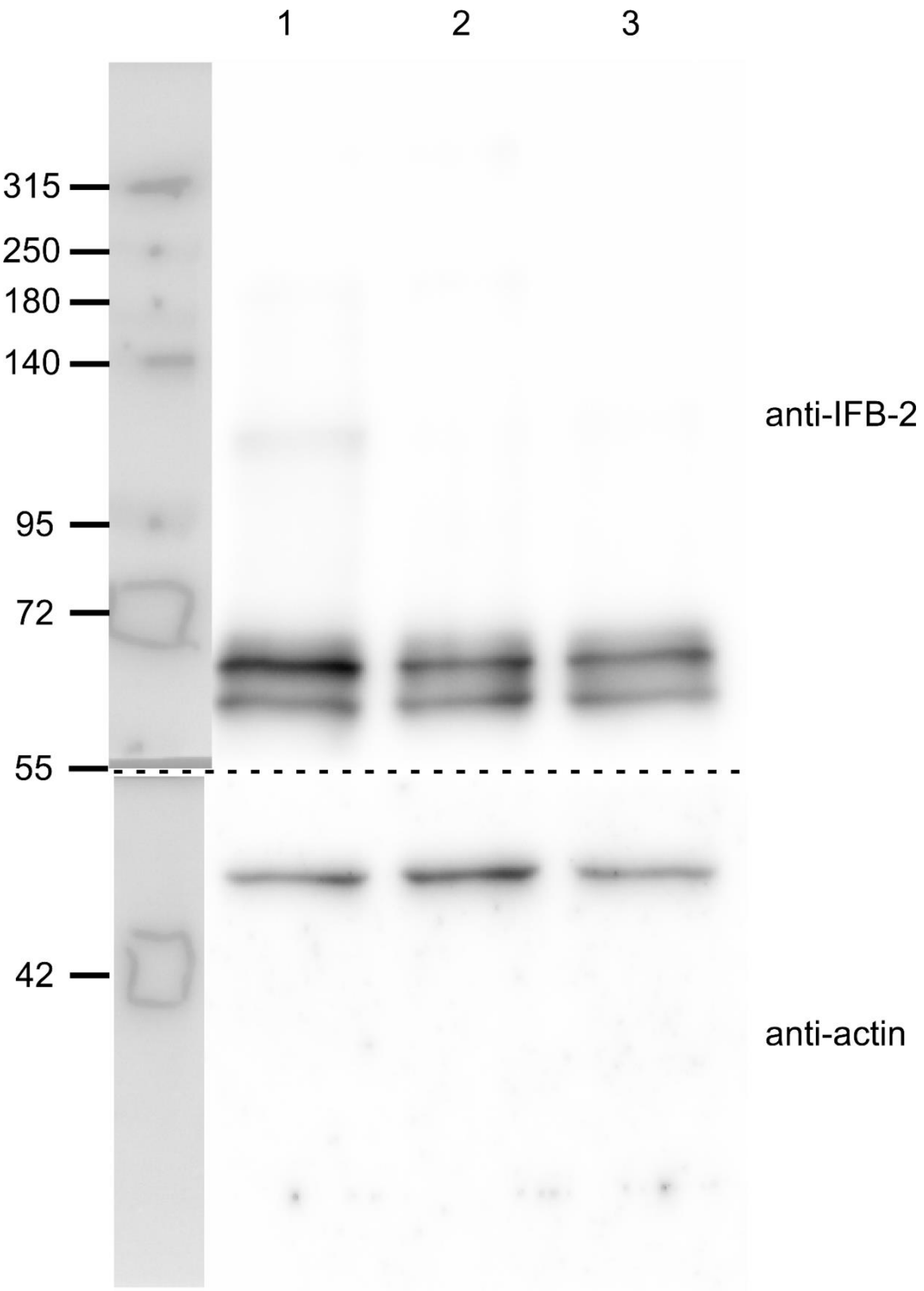

Supplementary Figure S8. Full-length blot referring to Figure 7g. The broken line indicates the position of the cutting line, which was generated to separate the upper part that was reacted with anti-IFB-2 antibodies and the lower part that was reacted with anti-actin antibodies. The corresponding co-electrophoresed and blotted marker lane is shown at left.

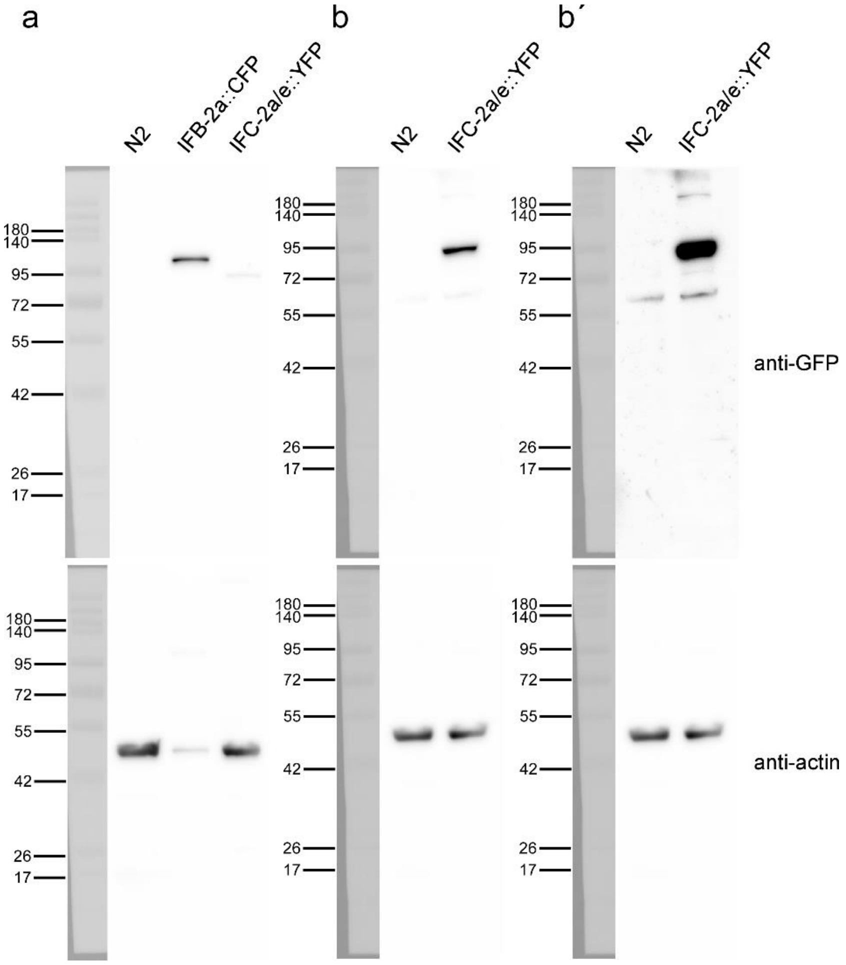

Supplementary Figure S9. Full-length blots referring to Supplementary Fig. S2 with corresponding co-electrophoresed and blotted marker lanes.

Supplementary Table S1. Strains used in this study.

STRAIN	GENOTYPE	Generation	SOURCE/REF	USE
N2	Wild type		CGC	Wild-type control
DP38	*unc-119(ed3)III*		CGC	Microinjection
BJ49	*kcIs6[ifb-2p::ifb-2a::cfp]IV*	Microinjection of plasmid *pifb-2::cfp* in N2 followed by UV-induced integration and 5x outcrossing with N2	[2]	Carboxyterminally tagged IFB-2::CFP reporter
BJ324	*kcEx78[ifc-1p::ifc-1::egfp; unc-119(ed3)+];unc-119(ed3)III*	Microinjection of fosmid clone 18076040888727174 F05 (transgeneome.mpi-cbg.de) in DP38	This study	Carboxyterminally tagged IFC-1::EGFP reporter
BJ316	*ifc-2(kc16[ifc-2a/e::yfp])X*	Microinjection of targeting plasmid #4110 and repair plasmid #4102 in N2 followed by screening for knockin animals in F2 and 6x outcrossing with N2	[3]	Carboxyterminally tagged IFC-2::YFP reporter
BJ145	*kcIs6[ifb-2p::ifb-2a::cfp]IV; ifc-2(kc16[ifc-2a/e::yfp]))X*	Cross of BJ49 with BJ316	This study	IFB-2::CFP/IFC-2::YFP double reporter
BJ251	*kcEx70[ifd-2p::ifd-2::egfp;unc-119(ed3)+];unc-119(ed3)III*	Microinjection of fosmid clone 095794875301489 H04 (transgeneome.mpi-cbg.de) in DP38	This study	Carboxyterminally tagged IFD-2::EGFP reporter
BJ312	*kcIs40[ifp-1p::ifp-1::egfp]IV*	Microinjection of fosmid clone 23762707929976346 G11 (transgeneome.mpi-cbg.de) in DP38 followed by X-ray induced integration and 3x outcrossing with N2	This study	Carboxyterminally tagged IFP-1::EGFP reporter
RB1742	*ifb-1(ok2227)II*		CGC	*ifb-1* knockout
BJ309	*ifb-2(kc14)II, kcIs7[ifb-2p::ifb-2a[40-41insT]::cfp] *kcIs6]IV*	Microinjection of knockout plasmid #4113 and selection plasmid pCFJ104 in BJ49 followed by screening for knockout animals in F2 and 4x outcrossing with BJ49	[3]	*ifb-2* knockout
BJ318	*ifc-2(kc15)X*	Microinjection of knockout plasmid #4123 and selection plasmid pCFJ104 in BJ316 followed by screening for knockout animals in F2 and 4x outcrossing with BJ316	This study	*ifc-2* knockout
BJ374	*ifd-1(ok2404)X*	Cross of strain ZB4795 (*ifd-2(bz187[del816275-815271]); bzIs166[Pmec-4::mCherry1]; ifd-1(ok2404)*) (from Monica Driscoll and Meghan Arnold) with N2	This study	*ifd-1* knockout
BJ375	*ifd-2(bz187)X*	Cross of strain ZB4795 (*ifd-2(bz187[del816275-815271]); bzIs166[Pmec-4::mCherry1]; ifd-1(ok2404)*) (from Monica Driscoll and Meghan Arnold) with N2	This study; Arnold et al., manuscript in preparation	*ifd-2* knockout

BJ320	*ifp-1(kc18)X;kcIs41[ifp-1p::ifp-1[16-19del]::egfp *kcIs40]X*	Microinjection of knockout plasmid #4135 and selection plasmid pCFJ104 in BJ312 followed by screening for knockout animals in F2	This study	*ifp-1* knockout
BK531	*exc-2(qp111[Pexc-2::gfp::3Xflag::exc-2])X*		[1]	Aminoterminally tagged EXC-2::GFP reporter
BK36	*qpIs11[vha-1p::GFP + unc-119(+)]I;unc-119(ed3)III*		CGC, [4]	VHA-1P::GFP reporter
BJ338	*ifc-2(kc15)X;qpIs11[vha-1p::GFP + unc-119(+)] I; unc-119(ed3)III*	Cross of BJ318 with BK36	This study	VHA-1P::GFP reporter in *ifc-2* knockout
RZB213	*plst-1(msn190[plst-1::gfp])IV*		[5]	Carboxyterminally tagged PLST-1::GFP reporter
BJ344	*plst-1(msn190[plst-1::gfp]) IV;ifb-2(kc14)II*	Cross of BJ309 with RZB213	This study	PLST-1::GFP reporter in *ifb-2* knockout
BJ359	*plst-1(msn190[plst-1::gfp]) IV;ifc-2(kc15)X*	Cross of BJ318 with RZB213	This study	PLST-1::GFP reporter in *ifc-2* knockout
	plst-1(tm4255)IV		[5]	*plst-1* knockout

Supplementary Table S2. Phenotype summary.

	ifo-1(kc2)	*sma-5(n678)*	*ifb-2(kc14)*	*ifc-2(kc15)*	*ifp-1(kc18)*
Endotube	Loss with junctional aggregates [6]	Discontinuous [7]	Complete loss [3, this study]	Thinning [this study]	n.d.
Lumen	Widening [6]	Multiple cytoplasmic invaginations and widening [7]	Occasional widening [this study]	Occasional widening [this study]	Normal [this study]
Microvilli	Highly disordered [6]	Highly disordered [7]	Slightly disordered [this study]	Rarefied [this study]	n.d.
Embryonic lethality	None [6,8]	None [7]	None [this study]	None [this study]	n.d.
Larval arrest	Yes [3,8]	Yes [3]	None [3, this study]	None [this study]	n.d.
Time of development	Increased [8]	Increased [7]	Slightly increased [3, this study]	Normal [this study]	n.d.
Brood size	Reduced [6,8]	Reduced [7]	Reduced [3, this study]	Reduced [this study]	n.d.
Life span	Reduced [8]	Reduced [7]	Increased [3, this study]	Reduced [this study]	n.d.
Cry5B-sensitivity					
- Larval arrest	100% [3]	82.09% [3]	88.41% [3, this study]	52.17% [this study, *]	n.d.
- Time of development	n.a. [3]	9.92 days [3]	8.77 days [3, this study]	8.10 days [this study]	n.d.
- Survival	-- [3]	- [3]	- [3, this study]	+ [this study]	n.d.
Oxidative stress					
- Survival	--- [3]	- [3]	- [3, this study]	--- [this study]	No difference to N2 [this study]
Osmotic stress					
- Lethality	+++ [3]	+ [3]	++ [3, this study]	++ [this study]	No difference to N2 [this study]

n.d., not determined; n.a., not applicable; *no difference to N2

References

1 Al-Hashimi, H., Hall, D. H., Ackley, B. D., Lundquist, E. A. & Buechner, M. Tubular Excretory Canal Structure Depends on Intermediate Filaments EXC-2 and IFA-4 in Caenorhabditis elegans. *Genetics* **210**, 637-652, doi:10.1534/genetics.118.301078 (2018).

2 Husken, K. *et al.* Maintenance of the intestinal tube in Caenorhabditis elegans: the role of the intermediate filament protein IFC-2. *Differentiation* **76**, 881-896, doi:10.1111/j.1432-0436.2008.00264.x (2008).

3 Geisler, F. *et al.* The intestinal intermediate filament network responds to and protects against microbial insults and toxins. *Development* **146**, doi:10.1242/dev.169482 (2019).

4 Mattingly, B. C. & Buechner, M. The FGD homologue EXC-5 regulates apical trafficking in C. elegans tubules. *Developmental Biology* **359**, 59-72, doi:10.1016/j.ydbio.2011.08.011 (2011).

5 Ding, W. Y. *et al.* Plastin increases cortical connectivity to facilitate robust polarization and timely cytokinesis. *J Cell Biol* **216**, 1371-1386, doi:10.1083/jcb.201603070 (2017).

6 Carberry, K. *et al.* The novel intestinal filament organizer IFO-1 contributes to epithelial integrity in concert with ERM-1 and DLG-1. *Development* **139**, 1851-1862, doi:10.1242/dev.075788 (2012).

7 Geisler, F. *et al.* A novel function for the MAP kinase SMA-5 in intestinal tube stability. *Mol Biol Cell* **27**, 3855-3868, doi:10.1091/mbc.E16-02-0099 (2016).

8 Geisler, F. *Identification and characterization of intestinal filament organizers in Caenorhabditis elegans.* PhD thesis, (2014).
